# Named Entity Recognition for Bacterial Type IV Secretion Systems

**DOI:** 10.1371/journal.pone.0014780

**Published:** 2011-03-29

**Authors:** Sophia Ananiadou, Dan Sullivan, William Black, Gina-Anne Levow, Joseph J. Gillespie, Chunhong Mao, Sampo Pyysalo, BalaKrishna Kolluru, Junichi Tsujii, Bruno Sobral

**Affiliations:** 1 School of Computer Science, University of Manchester, Manchester, United Kingdom; 2 National Centre for Text Mining, Manchester Interdisciplinary Biocentre, University of Manchester, Manchester, United Kingdom; 3 Virginia Bioinformatics Institute at Virginia Tech, Blacksburg, Virginia, United States of America; 4 Department of Computer Science, School of Information Science and Technology, University of Tokyo, Tokyo, Japan; Science Commons, United States of America

## Abstract

Research on specialized biological systems is often hampered by a lack of consistent terminology, especially across species. In bacterial Type IV secretion systems genes within one set of orthologs may have over a dozen different names. Classifying research publications based on biological processes, cellular components, molecular functions, and microorganism species should improve the precision and recall of literature searches allowing researchers to keep up with the exponentially growing literature, through resources such as the Pathosystems Resource Integration Center (PATRIC, patricbrc.org). We developed named entity recognition (NER) tools for four entities related to Type IV secretion systems: 1) bacteria names, 2) biological processes, 3) molecular functions, and 4) cellular components. These four entities are important to pathogenesis and virulence research but have received less attention than other entities, e.g., genes and proteins. Based on an annotated corpus, large domain terminological resources, and machine learning techniques, we developed recognizers for these entities. High accuracy rates (>80%) are achieved for bacteria, biological processes, and molecular function. Contrastive experiments highlighted the effectiveness of alternate recognition strategies; results of term extraction on contrasting document sets demonstrated the utility of these classes for identifying T4SS-related documents.

## Introduction

Named entity recognition (NER) research has focused on recognition of classes such as genes, proteins, and diseases. We explored recognition of less-studied classes of entities, such as cellular components and biological processes, to support enhanced access to the literature for users of the Pathosystems Resource Integration Center (PATRIC, patricbrc.org). We chose bacterial Type IV secretion systems (T4SSs) as our first area of focus with the intent of applying similar techniques in future work to other biological phenomena of interest to infectious disease researchers, such as pathogenicity mechanisms, virulence factors, colonization and incubation, and evasion of host immune response.

Searching literature related to T4SSs is difficult, in part, due to a lack of common terminology across bacterial species. In this introduction, we briefly describe bacterial T4SSs and their functional complexity, to demonstrate the extent of the synonym problem in this domain, and our approach to mitigate that problem with the use of named entity recognition techniques.

### Type IV Secretion Systems

At least seven distinct macromolecular translocation systems have been identified in prokaryotes for the transfer of molecules across intra- and intercellular barriers [1]. Currently, T4SSs are the only group of translocation machines that span the broad distribution of Prokaryota, being encoded within many genomes of both Gram negative and Gram positive species, as well as within some wall-less bacteria and Archaea [2]. Based on a survey of diverse subfamilies [3], it can be stated that T4SSs function predominantly in conjugation [4], naked DNA uptake and release [5], and the propagation of genomic islands [6]. As such, T4SSs are important factors in bacterial diversification and are responsible for the lateral mobilization of antimicrobial resistance and virulence genes. Additionally, T4SSs are also used by some bacterial species to transport effector molecules (DNA and/or protein) to eukaryotic host cells [7], a process that can facilitate infection and sometimes pathogenesis. For example, over 150 substrates of the dot/icm T4SS of *Legionella pneumophila* have been identified, many of which assist the bacterium in its avoidance of the host lysosomal network [8], [9]. Thus, given their broad phylogenetic scope, T4SSs encompass an extraordinary array of functional diversification and constitute a major player in infectious disease processes in many bacterial species. This level of biological complexity challenges their classification and characterization, yet because of their importance it is a worthwhile endeavor to do so.

**Figure 1:**
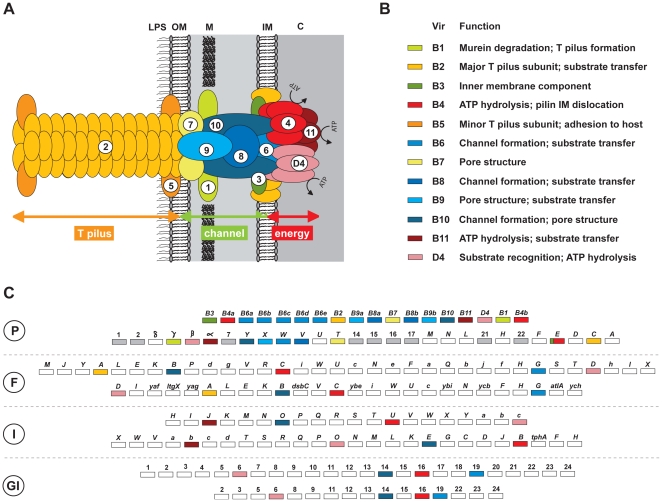
Complexity of Type IV secretion system (T4SS) architecture and nomenclature. (A) Model of the VirB/VirD P-T4SS encoded on the pTi plasmid of *Agrobacterium tumefaciens*. LPS  =  lipopolysaccharide, OM  =  outer membrane, M  =  murein layer, IM  =  inner membrane, C  =  cytoplasm. (B) Description of the VirB/VirD proteins. (C) Diversity encompassed by the major groups of T4SSs. P, P-T4SS: top  =  *Rickettsia prowazekii* (*rvh*) [31],[45] bottom  =  *Helicobacter pylori* (*cag* pathogenicity island, *cag*-PAI) [46]. Genes with homology to *vir* genes are colored accordingly. *cag*-PAI genes colored gray are not known to form the T4SS scaffold, while genes colored white are involved in T4SS function but have no clear homology to *vir* genes. F, F-T4SS: top  =  *Escherichia coli* (*tra*/*trb* of F plasmid), bottom  =  *Neisseria gonorrhoeae* (*tra*/*trb* of gonococcal genetic island). Capital letters depict *tra* genes while lower case letters depict *trb* genes, with remaining genes given their full names. I, I-T4SS: top  =  *tra*/*trb* of the IncI plasmid R64, bottom  =  *Legionella pneumophila* (*dot*/*icm*) [47]. Capital letters depict *icm* and *tra* genes while lower case letters depict *dot* and *trb* genes. GI, GI-T4SS: top  =  *Haemophilus influenzae* (*tfc*), bottom  =  *Salmonella enterica* Typhi (*tfc*). NOTE: Genes of F-, I- and GI-T4SSs with homology to *vir* genes are colored accordingly.

One confounding aspect of T4SSs pertains to gene nomenclature. Across the major groups of T4SSs, rarely are gene nomenclatural schemes consistent, even when informatics strongly supports orthology across these divergent families (Fig. 1). Relative to the archetypal *vir* T4SS, there exists a wide array of synonymous gene and protein names for components related to the *vir* genes. For example, VirB6 is synonymous with AvhB6, TrbL, Vbh6, CagX, TraG, Pfc19, and VblB6. In addition, T4SS function can be radically different across even closely-related species. While the *vir* T4SS of *A*. *tumefaciens*, which is essential for survival in its plant hosts and secretes a nucleoprotein complex into host cells that eventually results in insertion of tumerogenic DNA into the host genome [10], [11], the *vir* T4SS of closely-related *Sinorhizobium melliloti* is not essential for symbiosis with its host, but rather needed only for bacterial conjugation [12].

The diversity of terminology associated with genes and proteins across the range of organisms exhibiting these transport mechanisms can hinder unification of the related literature and knowledge, so we instead chose to focus on the organisms and mechanisms that define and describe the behavior of these T4SSs. Our hypothesis is that the introduction of information relating to cellular components, biological processes, molecular functions, and organisms will enable more robust identification of the literature in this and similar fields, when allied with existing well-developed systems for gene and protein recognition. Exploitation of such information, however, requires the creation of systems that can extract these novel classes of entities and concepts from the literature. Thus, we developed named entity recognition systems targeted to these new concept classes. We assessed their effectiveness not only with regard to a “gold standard” corpus that we created for training and evaluation, but also in terms of their ability to identify terminology which can distinguish T4SS-related documents from documents relating to other secretion systems and from more general documents in the biological domain.

### Related Research

The need for creation of controlled vocabularies targeted to microbiology has been noted in the literature [13]. While there are established ontologies, such as the Gene Ontology [14], there is a substantial disparity between the names and synonyms present in these ontologies and terminology used by authors. The text mining efforts described here aim to overcome these terminological barriers and can be used to aid ontologies such as GO by allowing GO to import synonyms revealed by the text mining effort and through the expansion of GO into key areas of interest to infectious disease researchers.

Many approaches have been applied to the NER problem in the biomedical domain, particularly in the area of gene/protein mentions [15], predominantly involving systems that exploit different types of machine learning techniques, such as Naive Bayes', Maximum Entropy, Support Vector Machines, and Conditional Random Fields [16], [17], [18], [19]. There has been little work published explicitly dealing with text mining targeting bacterial Type IV secretion systems. The terminology relevant to this domain spans several concept classes including: microorganisms, genes and proteins, and several concept classes from the Gene Ontology, notably cellular components, biological processes, and molecular function. In terms of potential pathogens, there has been some research on disease recognition [20], [21], [22], [23], [24], [25]. There has been very substantial research on recognition of genes and proteins, through several community evaluations, such as JNLPBA-2004 [26] and BioCreative [27], [28]. For cellular components, biological processes, and molecular function, most research has focused on assigning one or more GO tags to documents, as in GoPubMed [29]. While variants of these tasks have achieved classification accuracies just over 70%, a related BioCreative Challenge [27] indicated that due to lack of training data and the complexity of the task, no systems had yet achieved levels of accuracy sufficient for practical use. Our work targets the specific and more challenging task of recognizing the specific GO concept mentions associated with T4SS within the text.

## Materials and Methods

First, we describe the resources and techniques we applied to investigate the creation and application of named entity recognition systems for the T4SS domain. We begin by describing the methodology for identifying the entity types of interest. We then describe in detail the creation of a gold standard corpus for these entity classes for use in training and evaluation of our system. Next, we explain the extraction and tuning of terminological resources for this task, based on publicly available, large-scale, curated resources. Finally, we present the dictionary-based and hybrid machine-learning/dictionary-based approaches to named entity recognition employed in this work.

### Selection of Named Entity Classes

Ideally, a T4SS named entity recognizers would have been developed using an existing annotated corpus freely available to the text mining community; however, since recognizing T4SS entities represents novel challenges for text mining, there are no prior standard annotated corpora to serve as training data for machine learning algorithms or to provide a gold standard for evaluation. Furthermore, the types of relations and patterns of term occurrence that are interesting are not typically present in the abstracts, but more often appear as part of the full text of the articles. Therefore, we developed new training and evaluation corpus materials for these concepts of interest, based on annotation of full papers.

It was necessary to determine the types of entities that would be most useful for distinguishing T4SS-related documents. To facilitate this process, we used the term extraction service, TerMine (http://www.nactem.ac.uk/software/termine/) [30] to automatically recognize frequent multi-word terms in a corpus. The top terms identified by TerMine included “secretion system”, “Ti plasmid”, “outer membrane”, and “nuclear import.” Frequent single word terms could have been used as well although we believe single word terms such as “system”, “plasmid”, “membrane” and “nuclear” are less informative for the task at hand. TerMine was applied to 10 T4SS-related documents and 17 ‘near-miss’ documents; we refer to these documents as the Terminology Exploration Set. The positive training examples are selected from articles listed in a Type IV secretion system bibliography compiled by a domain expert. The bibliography is the basis for references in Gillespie [31] and includes 268 references (see Supporting Information S1). The set of negative examples was compiled by randomly selecting papers from journals on bacteriology, microbiology, cell stress, and other near miss topics. Less related topics, such as soil metagenomics and cancer, were included to ensure a broader coverage in molecular function and cell component areas. Two domain experts reviewed the list of negative examples; one paper was eliminated because it addressed Type IV secretion systems but referred to it as conjugation (a T4SS function). A list of the top-ranked 240 terms formed the basis for term category selection. The 20 highest ranked terms appear in Appendix A (included in Supporting Information S1).

### Corpus Creation and Annotation

We annotated the four concept classes that should aid in the identification of documents associated with Type IV Secretion Systems: bacteria names, cellular components, biological processes, and molecular functions.[32] For cellular components, biological processes, and molecular functions, we restricted the annotation, and subsequently recognition, to those subsets of the entity classes specifically linked to T4SS, as detailed below. Full annotation guidelines for all classes are available in the supplementary material, Supporting Information S1.

- Bacteria: Since T4SS are employed by bacteria to transport material we annotated all named instances of bacterial organisms. These names generally include genus and species, with the genus frequently abbreviated to its first letter. If present, subspecies, strain, and serovar names were annotated as part of the entity. For example, *Agrobacterium tumefaciens* str. C58 is annotated, as are its alternate forms, including *Agrobacterium tumefaciens* C58, *Agrobacterium tumefaciens*, and *A. tumefaciens*.
- Cellular components (as defined in GO [14]) were tagged if they were associated with Type 4 secretion systems. Association with GO terms was determined by a mapping from T4SS-related genes in UniProt to GO, described in detail in the Concept and Entity Recognition Resources section below. The same process was followed for biological process and molecular function classes. A list of primary associated concepts appears in Appendix B (see Supporting Information S1). Examples include: protein complex, membrane, and periplasmic space. In addition, terms whose concepts are related to the selected GO terms are also tagged, as are terms specific to conjugation apparatus, such as the sex pilus or conjugal pore.
- Biological Processes identified by GO and associated with T4SS were also tagged, along with related concepts with alternate lexical realizations. These concepts included: transport realized as protein transport process and conjugation also realized as conjugal transfer of DNA. When specific genes were included in the description of the process, they were also tagged, as in RP4 plasmid transfer. Finally, more general biological processes were tagged in a context of T4SS, including localization, translocation, and virulence.
- Molecular functions in GO were tagged if associated with T4SS. These functions include different types of binding, such as ATP binding or RNA binding, and classes of activity, such as hydrolase activity or protein transporter activity. Again, related concepts were tagged, as in GTP-binding.

Exact GO terms are rarely used in the text of publications so corpus annotators used their judgment to identify related terms when tagging the training corpus.

The corpus was seeded with 10 full text documents from the T4SS-targeted bibliography provided by a T4SS domain expert (described above). An exploratory manual annotation of full-text documents for bacteria names, cellular components, and biological processes was performed by a bioinformatician. Based on this initial annotation, only bacteria were recognized with good accuracy (>80%). For each of the latter three entirely novel concept classes, we created a more extensive annotated corpus through the use of NaCTeM's ‘accelerated annotation’ (Acela) interface Through this interface, the domain expert iteratively and interactively worked with the system to annotate candidate instances of an entity class, in a setting similar to active learning. We created an instance of the Acela system specific to each concept class, augmenting the original document set with a new set of untagged full-text documents, bringing the total set of documents to be annotated to 27, five of which are also present in the terminology exploration set. The domain expert used the interface to tag entity mentions until the system achieved an estimated coverage of over 95%, or until no additional positive instances were found. For quality control, another domain expert was asked to second-score a subset of the annotations for cellular component, a particularly complex class. Since the two domain experts had dissimilar annotations (inter-rater agreement score of F-measure = 42.1%), they worked together to create an adjudicated annotation and revised the guidelines for annotation. The detailed corpus statistics for the corpus are shown in Table 1 (Training and Test Corpus).

**Table 1:** Corpus statistics for T4SS concepts: Bacteria, Cellular Component (Cell. Comp.), Biological Process (Bio. Process.), Molecular Function (Molecular.Fn.).

	Fully Manual Annotation	Acela Annotation (with Manual Seeds)
# Documents	10	27
# Pseudo-sentences	2437	11914
# Tokens	63465	222966

	Bacteria	Cell. Comp.	Bio. Process	Molecular Fn
# Tagged Entities	526	2237	1870	203
# Tagged Tokens	1034	4440	3001	369

### Entity and Concept Recognition Resources

For each of the entity types, we constructed lexical resources tailored to the task from a combination of established, curated domain ontologies and term lists provided by domain experts. Detailed statistics for these resources appear in Table 2.

**Table 2:** Statistics for dictionaries extracted from domain-specific resources for each of the entity classes.

	Bacteria	Cell. Component	Bio. Process	Mol. Function
Full Ontology				
Head terms	100255	2451	17128	8655
Total entries	475612	4383	50566	31882
T4SS Branches				
Head terms	N/A	1418	2453	2880
Total entries	N/A	2766	5881	8369

All GO-related categories include terms extracted across the full Gene Ontology and for only the T4SS branches.

We merged two large-scale resources for scientific names for bacteria: the bacteria branch of the NCBI taxonomy (http://www.ncbi.nlm.nih.gov/Taxonomy) and the ‘List of Prokaryotic names with Standing in Nomenclature’ (LPSN, http://www.bacteria.cict.fr). We extracted all scientific bacteria names from these resources and converted them to a set of standardized forms that cover typical variability for these terms (see Supporting Information S1).

For the classes of cellular component, biological process, and molecular function, we extracted instances from the corresponding namespaces of the GO. The task-specific term list for biological processes, cellular components, and molecular functions was compiled with the following process. A domain expert compiled a list of 929 genes related to Type IV secretion systems. The list was developed based on the domain expert's understanding of the various names for the diverse array of Type IV secretion systems. For each gene, GO annotations were retrieved from UniProt. Domain experts reviewed the list to identify GO annotations specific to Type IV secretion systems that did not generally apply to other topics as well. A full list GO annotations retrieved from UniProt and the root T4SS concepts selected as relevant by domain experts can be found in Appendix C (see Supporting Information S1). From these root T4SS concepts, we extracted all names and synonymous forms for concepts on corresponding sub-branches of Gene to populate our dictionary resources. The reduction in terminology resulting from this T4SS domain-specific focus is highlighted in Table 2.

The output of the NER task is a tagged span of text identifying bacteria name or a concept that is a member of the set of concepts constituting the intersection of T4SS concepts and one of biological process, cellular component, or molecular function. For this task, the goal is to identify concepts related to T4SS at the top levels of the GO ontology. For evaluation purposes, a tagged span was considered correctly identified it contained a term related to T4SS,e.g. conjugation, and was in the context of a T4SS topic, e.g. bacterial conjugation but not molecular conjugation.

### Entity and Concept Recognition Approaches

We evaluated three recognition techniques: a pure dictionary approach, a dictionary plus corpus enrichment, and a machine learning approach. In a pure dictionary-based approach, matching is performed to identify the longest substrings, under simple orthographic normalization, that match in the static dictionaries created above. In the second strategy, dictionary-based matching with corpus enrichment, tagged terms found in a training portion of the corpus were added to the static dictionary and then matching was performed. This strategy allowed the system to augment the terminology found in standard domain resources with that present in running text.

The machine learning approach incorporated dictionary-based information and other features in a Conditional Random Field (CRF) tagger. CRFs are machine learning algorithms which take into account features of the context in which named entities appear. When used with natural language sentences, the words before and after a term constitute the context and features include the part of speech and capital/lower case patterns in those surrounding words. CRFs [33] have been used successfully to sequence labeling problems such as named entity recognition, part of speech tagging and parsing[34], [35]. The main advantage of CRFs is that they estimate the conditional probability distribution over labeled sequences and allow the information on the confidence of the decision to be used by other components in the text processing pipeline.[34], [35] We employed a linear chain CRF model trained on the annotated corpora converted to a standard named entity recognition data format. The format, known as the BIO format, labels each word in a text with a B, I or O for beginning of an entity, inside an entity, and outside and entity, respectively. For example, the term “transmission of DNA” is labeled as a biological process in the following phrase while the other words in the phrase are labeled as outside of a biological process term.

for      O

transmission  B

of      I

DNA     I

substrates   O

across    O

the     O

The machine learning component used three main sets of base features, inspired by previous research in biomedical NER [32]:

1. Lexical features included the current word, the root form of the current word, and the part-of-speech tag of the current word, computed by the Genia tagger [36].
2. Orthographic features comprised substring and word form features. In the word form features, all uppercase letters were normalised to ‘A’, lowercase to ‘a’, and all digits to ‘0’. The first two and four characters and last two and four characters of the original word and its normalised word form were chosen as features.
3. Dictionary features included binary features that indicate the presence of the word in our dictionary and the position of the word within any dictionary entries.

For each of the base features, corresponding features for words within a context window were added to the representation. The window ranged from 1–3 words preceding and following the current word.

## Results

### Entity Recognition

We performed recognition experiments across the four novel entity types targeted by the T4SS application domain: bacteria, cellular component, biological process, and molecular function. We explored three experimental contrasts: 1) simple dictionary-based tagging, 2) dictionary enrichment from a training segment of the tagged corpus, and 3) a hybrid dictionary-machine learning approach. For all training conditions, five-fold cross-validation was employed on a manually tagged corpus created specifically for this task. All results are presented are presented in terms of the standard metrics of precision, recall, and the harmonic mean of precision and recall, known as an F-measure. The results were computed with a version of the scoring script developed for the Conference on Natural Language Learning (CoNLL)-2000workshop evaluation [37] and The CoNLL-2000 workshop assessed system performance on a ‘chunking’ task, involving finding phrases in text. By analogy, our current task can be viewed as finding entity phrases in text, making this program suitable for analysis. The results of these experiments appear in Table 3 below.

**Table 3:** Entity Recognition across classes contrasting dictionary-based, dictionary-based with corpus enrichment, and machine learning strategies.

	Bacteria			Cellular Comp.			Biological Proc.			Molecular Fun.		
# Entities	526			2237			1870			203		
	P	R	F	P	R	F	P	R	F	P	R	F
Dictionary	96	97	96	50	11	18	59	35	44	64	62	63
Dictionary+Corpus	96	97	97 fsd(8)	49	59	54 (701)	66	86	75 (366)	69	83	75 (71)
Machine Learning	93	91	93	74	62	68	87	81	84	92	82	86

Abbreviations are as follows: P = precision, R = recall, and F = F-measure, the harmonic mean of precision and recall. The number of distinct terms added by corpus enrichment is given in parentheses.

Results ranged from F-measures of 18% to 96% for pure dictionary-based approaches, from 54% to 97% for dictionary-based approaches with dictionary enrichment from corpus, and from 68% to 93% for machine learning methods, using all features. Tables 3 and 4 show some interesting contrasts. In the cases of cellular component and biological process, pure dictionary-based results were quite poor, corpus enriched dictionary results showed substantial improvement, and machine learning results ranged from fair (cellular component) to excellent (biological process, molecular function). This contrast is consistent with the fact that the basic terminology found in GO, from which the dictionaries for these classes were selected, is frequently unlike that which is found in typical published scientific text. This contrast was also highlighted by the annotators themselves and was reflected in the annotation guidelines. As a result, inclusion of tagged terms from the annotated corpus with the domain dictionaries introduces the more common term variants, and machine learning techniques enable further generalization and disambiguation.

Conversely, pure dictionary-based approach for the annotation of Bacteria yielded the best results overall, and an extremely high recall rate of 97%. This suggests that the normalized term list extracted for bacteria was well-matched to this task, capturing most sources of variability. The relatively poorer accuracy for the machine learning approach in this case indicates that some small remaining inconsistencies in tagging may be introducing some noise into the training process that misleads the probabilistic learner.

### Using Terms to Identify T4SS-related Documents

This investigation of the recognition of these novel entity classes was motivated by their potential to distinguish documents associated with T4SS from documents which are not, a common and difficult task faced by annotators working in large-scale bioinformatics resources such as PATRIC. Using the entity recognition systems developed above, we compared the use of this terminology across three different document classes: T4SS documents (10 documents from the terminology exploration set), ‘near-miss’ documents (17 documents also from the terminology exploration set), and an additional 10 general non-T4SS documents, drawn from various journals based on broad searches such as ‘soil metagenomics’ and ‘cancer’. For the documents in each of these classes, we applied the best-performing automatic concept recognition approach for each concept category determined by the earlier experiments. We then extracted all instances of recognized phrases and ranked them by frequency of occurrence within each of the document classes. Table 4 contrasts the distribution of these entity classes across the document classes.

**Table 4:** Number of terms in each class for Bacteria, Cellular Component, Biological Process, and Molecular Function classes for T4SS, near-miss, and general documents.

	T4SS Documents	‘Near-miss’ Documents	General
Bacteria	230	259	30
Cellular Components	208	92	48
Biological Process	215	160	58
Molecular Function	20	13	4

Numbers are scaled by corpus size for each class.

These results indicate, as expected, dramatic differences in the distribution of T4SS-associated terminology across the document classes. T4SS-related terms occur in T4SS documents at 4–8 times the rate they appear in the general document class. With the exception of bacteria, the T4SS-related terms are still found in T4SS documents at 1.3 to 2 times their rate of occurrence in near-miss documents. Since the near-miss documents refer to secretion systems, though not Type IV, which are themselves observed in bacteria, it is not surprising that bacteria mentions appeared at a similarly high rate for both T4SS and near-miss documents.

The differences in the mechanisms of different types of secretion systems are further highlighted in the specific terms employed and the differences in term distributions. Only, 7% (cellular component) to 20% (bacteria) of the distinct terms recognized in each of the concept classes appear in both T4SS and near-miss documents. For example, terms relating to conjugation, T-complex, and dot/icm transporter genes were strongly associated with T4SS documents as were bacteria that exhibit these systems. These terms, however, appeared infrequently or not at all in the concepts tagged in even the near-miss documents and much less the general documents. In contrast, general secretion terms and terms that were strongly associated with other specific types of secretion systems were recognized frequently in the near-miss documents. These terms associated with other secretion systems, such as bacteria (e.g., *Yersinia* and *Pseudomonas*) and cellular components (e.g., cytosol), were strongly associated with these documents, while remaining infrequent or absent in the T4SS documents. These strong contrasts in the distribution across the three document classes of recognized concepts in these T4SS-associated classes support the utility of these terms for automatic classification and recognition of T4SS documents.

## Discussion

### Impact of Corpus-enrichment

With the exception of the bacteria class, the use of corpus-based enrichment dramatically improves NER effectiveness over the fixed dictionary. In the case of bacteria, the dictionary has near-exhaustive coverage of the domain and has been automatically expanded with standard variant forms of the terms. Furthermore, bacterial scientific names are minimally inflected, with only singular and plural forms, and they are rarely abbreviated except for the initial of the genus when the binomial is used. As a result, term coverage is very high, and only eight forms are added.

For cellular components and biological processes, a fairly large number of term forms are added (366 to 701, though many of the latter differ only in minor formatting) in contrast with the small number of terms added for the bacteria class. This difference indicates both the better coverage of the bacteria term resources and the greater degree of variability of the expressions used for the other classes. The relatively small number of terms added to the molecular function class results from the combination of the small number of tagged instances (less than 200) and a highly restricted class that includes a restricted set of types of binding and activity classes. The vast majority of the terms added (86% for biological process) are paraphrase variants of each other and of entries in the dictionary, often with further restriction through arguments or modifiers. These forms are fairly consistent but only moderately productive, so it would be problematic to attempt to generate all such forms exhaustively for a dictionary-based system. In contrast, the machine learning approaches can automatically acquire these general patterns to support robust recognition. Below we analyze this variability in greater detail and present strategies to manage it through entity mention normalization.

### Analysis of Entity Mention Variability

An understanding of the ways in which entities of interest are referred to in text is necessary for the efficient development of methods for detecting entity mentions and for mapping detected mentions to specific database or ontology identifiers. To analyze the variation of entity mentions in the T4SS corpora, we applied normalization methods to the four sets of annotated gold standard mentions, measuring the efficiency of each normalization approach in reducing the number of unique text strings in each of these sets. We applied standard normalizations addressing typographical, morphological, and syntactic variation, applied some partially domain-specific term reduction rules, and resolved abbreviations.

Typographical variation was addressed by string matching that ignores, for example, capitalization and hyphenation in spelling variants, and morphological variation by lemmatization, that is, restoring each word of an entity mention to its word root. As the annotated entity mentions contained only little syntactic structure, a small number of hand-written rules were used to normalize basic syntactic variants such as alternation between noun-modifying and prepositional phrase variants of a term, e.g. “DNA binding” vs. “binding of DNA”. Normalization drawing on the semantics of the annotated words was limited to the reduction of semantically light head words,e.g. “activity”, when the removal of such words allowed a match with another annotated term. For abbreviations, a local acronym dictionary was extracted from definitions found in the corpus, and different shortened, full, and abbreviation-defining forms were replaced with a standard canonical form of each acronym. Finally, a single rule implementing the common form of abbreviating species names, e.g. *Escherichia coli* vs. *E. coli*) was applied in cases where the abbreviation matched another annotated entity. Examples of each of these types of variation are given in Table 5.

**Table 5:** Typological breakdown of entity mention variability in typographical, morphological, syntactic, reduction, and abbreviation classes.

	Examples
Typographical	Nucleotide binding, nucleotide-binding, NUCLEOTIDE-BINDING
Morphological	localize, localizes, localized, localization
Syntactic	DNA translocation, translocation of DNA, translocates DNA
Reduction	secretion process, secretion/ATP-binding activity, ATP-binding
Abbreviations	type IV secretory system, T4SS,Type IV secretion system (TFSS)

**Figure 2:**
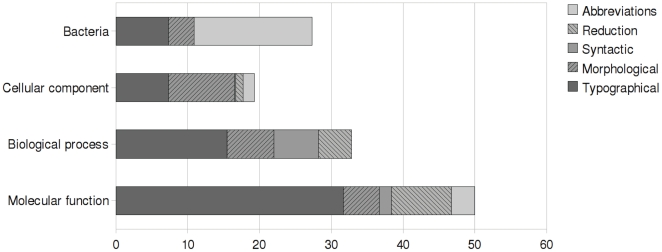
Comparison of the effect of normalization. Different classes of entity mention variability (Typographical, Morphological, Syntactic, Reduction, and Abbreviation) across different entity classes (Bacteria, Cellular component, Biological process, and Molecular function). The graph indicates the percentage reduction in unique strings contributed by each class of normalization process.

Table 6 shows the effect of the normalization on the number of unique strings in each of the annotated entity classes, showing a notable decrease for all classes. The effectiveness of the different normalization strategies varied considerably by class (Figure 2). For bacteria, we find limited typographical and morphological variation and no syntactic variation or head words that could be reduced, likely reflecting the rigidity of the species' names. By contrast, for classes other than bacteria, we find significant typographical and morphological variation – together accounting for the majority of all variation – as well as notable benefit from syntactic normalization (esp. for biological process) and reduction (esp. for molecular function). While the resolution of abbreviations contributes to normalization for all classes except biological process, the effect is most significant for bacteria, reflecting the frequency of occurrences of abbreviated species' names.

**Table 6:** Impact of normalization of entity mentions expressed by reduction in number of unique strings, broken down by entity class.

	Original	Normalized	Decrease
Bacteria	55	40	27%
Cellular Component	698	563	19%
Biological Process	323	217	33%
Molecular Function	60	30	50%

The results of this analysis largely agree with our previous studies of biomedical domain terminology [30], [38], demonstrating that the variation found in T4SS terms falls largely under types addressed by previously introduced methods for e.g. soft string matching [39], lemmatization [40], and acronym detection and resolution [41]. Future efforts will include the automation of T4SS term normalization through the use of these methods and integration of the recognition and normalization into search functionality in tools such as Medie [42].

### Entity Contrasts

Unlike much prior work on Named Entity Recognition (NER), we do not aim to recognize all members of an ontological class, but instead selectively recognize task-specific subsets of broader ontological classes. Rather than recognizing all cellular components, for instance, we aim to identify only those entities, and their subclasses, linked to ontological concepts mapped from entities in UniProt, associated with T4SS and more general terminology applied to T4SS contexts. This perspective emphasizes precision in entity and concept recognition. This focus on precision, filtering of terms and contexts associated with T4SS, provides an advantage to machine learning techniques which can exploit these contextual restrictions.

In addition, the types of features which provide evidence for recognition of these new entity classes also differ from those which have typically been observed for more commonly studied Named Entities, such as genes and proteins. The terms added by corpus enrichment highlight some of these contrasts. For example, word shape, such as patterns of capitalization and digits, has often been identified as a key feature in entity recognition for gene and protein mentions. However, among these entities, only bacteria have highly consistent orthographic cues, as in ‘*A. tumefaciens*’ where the pattern of capital letter, dot, space, and lowercase term is a strong cue to Latinate organism names, though this does not distinguish among organism classes easily. Strain names likewise may be cued by orthographic patterns. No such patterns appear for biological processes, and only in plasmid names for cellular components.

### Contrasting approaches through error analysis

In comparing the errors made by the different approaches, we observed some consistent patterns. In the pure dictionary approaches, phrasal variants are missed, and all instances of dictionary terms were tagged. The first lowered recall and the second reduced precision. For example, in the case of ‘transfer’ from above, neither that term nor any of its labeled phrasal variants were present in the original dictionary, although over 350 such instances are annotated in the corpus. Clearly, this had a severe deleterious effect on recall. In the case of cellular components, ‘plasmid’ followed a similar pattern. Conversely, ‘transport’ is in the dictionary, and while it was present in over 140 of the labeled biological process instances, it also appeared over 71 times unlabeled, where non-T4SS transport was described or where it specified a cellular component instead. Since all instances were labeled by the dictionary-based approach, this significantly degraded precision. Many other terms exhibited similar patterns, including ‘binding’ for molecular function. This behavior was prevalent across the GO concept classes and results in their poor performance. In contrast, for bacteria, variation was more regular and was adequately captured by the dictionary creation process, yielding few errors.

Corpus-enrichment significantly mitigated the problem of missing phrasal variants that reduce recall. However, it can introduce severe problems of over-generalization that can exacerbate the problems with precision. For example, a single annotated instance of the term ‘formation’ caused the corpus-enriched dictionary-based system to tag all subsequent mentions, incorrectly as it turns out. A similar problem arose for the term ‘transfer’.

Machine learning exhibited improved precision for all classes, with dramatic improvements for the GO-related concept classes. It yielded only small reductions in recall in most cases, with the exception of the small improvement found for cellular components. This effectiveness can be attributed largely to two factors: the use of contextual features by the machine learning system and the use of probabilistic evidence. While the dictionary-based approaches tagged all and only those terms in their current working dictionary, machine learning approaches employed probabilistic classification based on the observed contextual training examples. The same term may be tagged differently by the system in different positions in the documents, based on the context of appearance. These systems did not exhibit the extreme over-generalization of dictionary-based approaches given a single term mention. For many terms, only a small proportion of their mentions should actually be tagged, penalizing the recall-oriented dictionary approaches and improving the more precision-oriented machine learning approach. As a result, these approaches perform well across all entity classes.

### Future Work

Although the focus of this paper has been on the specific task of named entity recognition for the key entity classes associated with T4SSs, our future plans emphasize the application of this component technology to enhance semantic search and information extraction, both broadly and for this specific domain of interest. We will deploy the NER techniques developed in this work to enhance the large-scale semantic search system, KLEIO (38) (http://www.nactem.ac.uk/software/kleio/) developed at NaCTeM (http://www.nactem.ac.uk). This system provides concept-based, rather than keyword-based, retrieval, highlighted display of named entities within retrieved abstracts, and faceted search based on the indexed classes of named entities. The inclusion of additional entity classes, such as bacteria, will further enrich this system for the community.

In future phases of the collaboration between PATRIC and NaCTeM, we will build on this work in three additional ways. First, we will leverage the NER built in this work, as well as other NaCTeM text mining tools and services, to support information extraction tasks that exploit these domain resources. In particular, we plan to mine relationships involving genes and proteins of bacterial pathogens, supported by the new bacteria recognizer,existing gene/protein recognition systems, and other tools from NaCTeM, such as [43], [44]. We will also incorporate this advanced entity recognition into PATRIC. Finally, we will apply similar techniques to other biological phenomena of interest to infectious disease researchers, such as pathogenicity mechanisms, virulence factors, colonization and incubation, and evasion of host immune response. In this way, we will create a highly functional and adaptable portal by adding text mining functionality to the PATRIC system. Through a plug-in architecture, we will be able to incorporate an expanding range of new text mining-based capabilities, encompassing named entity recognition across diverse entities and detailed relation and event extraction.

A web demonstrator is available for testing the named entity recognizer developed in this work at NaCTeM's portal http://www.nactem.ac.uk/T4SS_NER/top.py and http://patricbrc.vbi.vt.edu/portal/portal/patric/NACTEM. Lexical resources are available at these sites as well.

The Supporting Information S1 and S2 files include a set of appendices and compressed file with instructions and data for generating T4SS NERs. The seven appendices are as follows. Appendix A is a list of the top twenty multi-word terms identified in the T4SS documents. Appendix B is the set of GO terms related to T4SS. Appendix C contains a list of GO terms retrieved from UniProt using a list of 929 T4SS genes. Appendix D describes an assessment of the stability of the annotation process and the impact of errors in annotation. Appendix E lists the most frequent terms for Bacteria, Cellular Component, Biological Process, and Molecular Function classes for T4SS, near-miss, and general documents. Appendix F is a bibliography of T4SS literature. Appendix G is a set of guidelines used by annotators when tagging the training documents.

## Supporting Information

Supporting Information S1 Supplementary Material
(0.16 MB DOC)

Supporting Information S2 Source document extracts and scripts. The results described in this paper can be recreated by following the workflow described in the file entitled "Instructions for Executing T4SS Named Entity Recognition Workflow.docx."
(4.93 MB ZIP)
