# Supplementary material for: Named Entity Recognition for Bacterial Type IV Secretion Systems
Source: PLoS One. 2011 Mar 29;6(3):e14780. doi: 10.1371/journal.pone.0014780 (PMC3066171; doi:10.1371/journal.pone.0014780)
Supplement: Supporting Information S1 — Supplementary Material (0.16 MB DOC) [file pone.0014780.s001.doc]

# S1 Supporting Information

# Appendix A: Top 20 terms identified by TerMine in T4SS documents

secretion system

Agrobacterium tumefaciens

Ti plasmid

VirB protein

VirB operon

T-DNA transfer

vir gene

outer membrane

host cell

A. tumefaciens

vir protein

nuclear import

H. pylori

Helicobacter pylori

virulence gene

VirB promoter

VirB11 protein

membrane protein

Agrobacterium tumefaciens Ti plasmid

virulence protein

# Appendix B: T4SS-related Components in GO

T4SS-related Cellular Components in GO:

GO:0032991 macromolecular complex

GO:0043234 protein complex

GO:0043684 type IV secretion system complex

GO:0009279 cell outer membrane

GO:0016021 integral to membrane

GO:0016020 membrane

GO:0019867 outer membrane

GO:0005886 plasma membrane

GO:0042597 periplasmic space

T4SS-related Biological Processes in GO:

GO:0030030 cell projection organization

GO:0016998 cell wall macromolecule catabolic process

GO:0051716 cellular response to stimulus

GO:0033554 cellular response to stress

GO:0000746 conjugation

GO:0000747 conjugation with cellular fusion

GO:0042742 defense response to bacterium

GO:0009405 pathogenesis

GO:0009253 peptidoglycan catabolic process

GO:0009297 pilus assembly

GO:0009306 protein secretion

GO:0033103 protein secretion by the type IV secretion system

GO:0050896 response to stimulus

GO:0006950 response to stress

GO:0032940 secretion by cell

GO:0006810 transport

GO:0009291 unidirectional conjugation

T4SS-related Molecular Functions in GO:

GO:0005488 binding

GO:0005524 ATP binding

GO:0003676 nucleic acid binding

GO:0003677 DNA binding

GO:0003684 damaged DNA binding

GO:0003697 single-stranded DNA binding

GO:0003723 RNA binding

GO:0000166 nucleotide binding

GO:0017076 purine nucleotide binding

GO:0016787 hydrolase activity

GO:0003796 lysozyme activity

GO:0017111 nucleoside-triphosphatase activity

GO:0004386 helicase activity

GO:0008565 protein transporter activity

# Appendix C: GO Terms Returned from UniProt

The following terms were retrieved from UniProt using a list of 929 T4SS genes identified by Joe Gillespie. Only the terms highlighted in green (terms from expert 1) or yellow (terms from expert 2) were used in the T4SS NER dictionary.

929 results for job:TS5E in uniprot browsing by go

NOTE: light green highlighting suggests terms that are relevant to T4SS and not too general - JJG

* biological process 381

o biological regulation 11

+ cell redox homeostasis 1

+ regulation of biological process 11

# regulation of cellular process 10

* cell redox homeostasis 1

* regulation of cellular metabolic process 9

o regulation of nucleobase, nucleoside, nucleotide and nucleic acid metabolic process 9

+ regulation of transcription 9

# regulation of transcription, DNA-dependent 8

+ regulation of transcription, DNA-dependent 8

o regulation of transcription 9

+ regulation of transcription, DNA-dependent 8

# regulation of metabolic process 10

* regulation of biosynthetic process 9

o regulation of transcription 9

+

* regulation of cellular metabolic process 9

o

* regulation of macromolecule metabolic process 9

o regulation of transcription 9

+

o regulation of transcription, DNA-dependent 8

* regulation of primary metabolic process 10

o regulation of carbohydrate metabolic process 1

o regulation of nucleobase, nucleoside, nucleotide and nucleic acid metabolic process 9

+

o cellular component organization 8

+ cell projection organization 8

# pilus assembly 8

+ pilus assembly 8

o cellular process 22

+ cell projection organization 8

#

+ cell redox homeostasis 1

+ cellular metabolic process 8

# aminoglycan metabolic process 1

* glycosaminoglycan metabolic process 1

o peptidoglycan catabolic process 1

* peptidoglycan catabolic process 1

# cellular biosynthetic process 2

* cobalamin biosynthetic process 1

* DNA replication 1

# cellular carbohydrate metabolic process 1

* aminoglycan metabolic process 1

o

* peptidoglycan catabolic process 1

# cellular macromolecule metabolic process 6

* cellular biopolymer metabolic process 5

o DNA metabolic process 4

+ DNA repair 3

+ DNA replication 1

o DNA replication 1

o mRNA metabolic process 1

* DNA replication 1

* peptidoglycan catabolic process 1

# cofactor metabolic process 1

* cobalamin biosynthetic process 1

* porphyrin metabolic process 1

o cobalamin biosynthetic process 1

# electron transport chain 1

# nucleobase, nucleoside, nucleotide and nucleic acid metabolic process 5

* DNA metabolic process 4

o

* mRNA metabolic process 1

# peptidoglycan catabolic process 1

# tetrapyrrole metabolic process 1

* cobalamin biosynthetic process 1

* porphyrin metabolic process 1

o

# vitamin metabolic process 1

* cobalamin biosynthetic process 1

* water-soluble vitamin metabolic process 1

o cobalamin biosynthetic process 1

+ cellular response to stimulus 3

# cellular response to stress 3

* DNA repair 3

* SOS response 1

# SOS response 1

+ cytolysis 1

+ secretion by cell 6

# protein secretion 6

*

# protein secretion by the type IV secretion system 4

+ SOS response 1

o conjugation with cellular fusion 81

o cytolysis 1

o establishment of localization 130

+ protein secretion 6

# protein secretion by the type IV secretion system 4

+ secretion by cell 6

#

+ transport 130

# protein secretion 6

*

# secretion by cell 6

*

o localization 130

+ establishment of localization 130

#

+ protein secretion 6

#

+ secretion by cell 6

#

o metabolic process 9

+ biosynthetic process 2

# cellular biosynthetic process 2

*

# cobalamin biosynthetic process 1

# macromolecule biosynthetic process 1

* DNA replication 1

+ catabolic process 1

# carbohydrate catabolic process 1

* peptidoglycan catabolic process 1

# cell wall macromolecule catabolic process 1

# macromolecule catabolic process 1

* peptidoglycan catabolic process 1

# peptidoglycan catabolic process 1

+ cellular metabolic process 8

#

+ electron transport chain 1

+ glycerol ether metabolic process 1

+ macromolecule metabolic process 6

# biopolymer metabolic process 6

*

# cell wall macromolecule catabolic process 1

# cellular macromolecule metabolic process 6

*

# macromolecule biosynthetic process 1

*

# macromolecule catabolic process 1

*

+ nitrogen compound metabolic process 6

# aminoglycan metabolic process 1

*

# nucleobase, nucleoside, nucleotide and nucleic acid metabolic process 5

*

+ primary metabolic process 6

# carbohydrate metabolic process 1

* carbohydrate catabolic process 1

o

* cellular carbohydrate metabolic process 1

o

* polysaccharide metabolic process 1

o aminoglycan metabolic process 1

+

o peptidoglycan catabolic process 1

# nucleobase, nucleoside, nucleotide and nucleic acid metabolic process 5

*

o multi-organism process 318

+ conjugation 214

# conjugation with cellular fusion 81

# unidirectional conjugation 119

+ defense response to bacterium 1

+ pathogenesis 118

+ unidirectional conjugation 119

o pilus assembly 8

o response to stimulus 4

+ cellular response to stimulus 3

#

+ defense response to bacterium 1

+ response to stress 4

# cellular response to stress 3

*

# defense response to bacterium 1

# DNA repair 3

+ SOS response 1

* cellular component 226

o cell outer membrane 11

o cell part 225

+ cell outer membrane 11

+ cytoplasm 56

+ external encapsulating structure 11

# cell outer membrane 11

+ integral to membrane 58

+ intracellular 102

# cytoplasm 56

+ membrane 123

# integral to membrane 58

# outer membrane 19

* cell outer membrane 11

# plasma membrane 69

+ periplasmic space 2

GO:0043684 : type IV secretion system complex

o cell part 225

GO:0043684 : type IV secretion system complex

o extracellular region 1

o GO:0032991 : macromolecular complex

GO:0043234 : protein complex [11833 gene products]

GO:0043684 : type IV secretion system complex

* molecular function 202

o binding 195

+ ATP binding 186

+ nucleic acid binding 10

# DNA binding 8

* damaged DNA binding 1

* single-stranded DNA binding 1

# RNA binding 1

+ nucleotide binding 186

# ATP binding 186

# purine nucleotide binding 186

* ATP binding 186

+ transcription factor binding 5

o catalytic activity 77

+ hydrolase activity 72

# cobyrinic acid a,c-diamide synthase activity 1

# lysozyme activity 1

# nuclease activity 2

* endonuclease activity 1

* exodeoxyribonuclease V activity 1

* exonuclease activity 1

o exodeoxyribonuclease V activity 1

# nucleoside-triphosphatase activity 68

* helicase activity 1

+ protein disulfide oxidoreductase activity 2

+ transferase activity 2

# DNA-directed DNA polymerase activity 1

# NAD+ ADP-ribosyltransferase activity 1

o electron carrier activity 1

o protein transporter activity 2

# Appendix D: Assessing stability of annotations

### To understand the stability of the annotation process, and the impact of errors in annotation, we compare the automatic term recognition levels achieved prior to final annotation adjudication to the results obtained after this careful analysis process. The results of entity recognition prior to final annotation adjudication are computed for the four entity classes - bacteria, cellular components, biological processes, and molecular functions – for each of the three recognition approaches – dictionary-based, dictionary-based with corpus enrichment, and hybrid dictionary-machine learning (Table D1). Compared to the results in Table 3, we see a small to modest consistent decrease in effectiveness for most of the GO concept –related classes of 2% -8% for most measures for all NER approaches. This drop reflects a small number of inconsistent or erroneous annotations in the unadjudicated gold standard. For these classes, inspection of the changes in annotation shows that slightly more annotations are present in adjudicated annotations, but with no particular pattern. In contrast, these unadjudicated annotations show substantially lower performance for the bacteria class. Here, the errors in the original annotation had clustered, with several Vir terms incorrectly included and two bacteria name classes missed. The correction of these more consistent errors by the adjudication led to a larger overall improvement. The somewhat unexpected decrease in recall for bacteria in the corpus-enrichment setting is due to an incorrect annotation of a longer phrase, which blocks the correct subphrase from being recognized by the system.

**Table D1. Entity Recognition *prior to adjudication of annotations* across classes contrasting dictionary-based, dictionary-based with corpus enrichment, and machine learning strategies. Abbreviations are as follows: P is precision, R is recall, and F is the F-measure, the harmonic mean of precision and recall. Number of distinct terms added by corpus enrichment is given in parentheses.**

|  | Bacteria | | | Cellular Comp. | | | Biological Proc. | | | Molecular Fun. | | |
| --- | --- | --- | --- | --- | --- | --- | --- | --- | --- | --- | --- | --- |
| # Entities | 484 | | | 1913 | | | 1851 | | | 196 | | |
|  | P | R | F | P | R | F | P | R | F | P | R | F |
| Dictionary | 83 | 91 | 87 | 49 | 11 | 18 | 58 | 34 | 43 | 56 | 56 | 56 |
| Dictionary+  Corpus | 65 | 88 | 75  (31) | 45 | 59 | 51  (695) | 65 | 85 | 73  (374) | 60 | 77 | 67  (60) |
| Machine  Learning | 81 | 76 | 78 | 72 | 55 | 62 | 84 | 78 | 83 | 93 | 78 | 85 |

# Appendix E: Most frequent terms for Bacteria, Cellular Component, Biological Process, and Molecular Function classes

Table E1: Most frequent terms for Bacteria, Cellular Component, Biological Process, and Molecular Function classes for T4SS, near-miss, and general documents. Number of terms in each class, scaled by corpus size for each class, precedes the list of terms.

| Bacteria | | | |
| --- | --- | --- | --- |
| T4SS | Near-Miss | | General |
| 230 | 259 | | 30 |
| *Agrobacterium*  *Agrobacterium tumefaciens*  *H. pylori*  *Legionella pneumophila*  *A. tumefaciens*  *L. pneumophila*  *Helicobacter pylori*  *Legionella*  *E. coli*  *Salmonella* | *Yersinia*  *Escherishia coli*  *Salmonella*  *Pseudomonas aeruginosa*  *P. aeruginosa*  *E. coli*  *S. typhimurim*  *Salmonella typhimurim*  *Yersinia spp.*  *Yersinia enterocolitica* | | *E. coli*  *Streptomyces*  *H. polymorpha*  *Streptomyces lidivans*  *S. lidivans*  *Escherichia coli* |
| Cellular Components | | | |
| T4SS | Near-Miss | | General |
| 208 | 92 | | 48 |
| Type IV systems  Cytoplasmic membrane  T-complex  T-DNA transfer system  Pili  Outer membrane  Conjugation systems  Ti plasmid  T-complex transport Apparatus  Dot/Icm transporter | Outer membrane  Cytoplasm  Cytoplasmic membrane  Cytosol  Periplasm  Cell membrane  Inner membrane  Plasma membrane  Secretion apparatus  Secretion system | | Peroxisome  Cytoplasm  Pore  Nuclear pore complex  Peroxisomal membrane  Membrane  Lipid bilayer  Chromosome  Biotin system  Translocation complex |
| Biological process | | | |
| T4SS | Near-Miss | | General |
| 215 | 160 | | 58 |
| Virulence  Conjugation  Transfer  Export  Nuclear import  Assembly  T-DNA transfer  Secretion  DNA transfer  Translocation | Secretion  Virulence  Translocation  Secreted  Export  Assembly  Transport  Protein secretion  Secretion pathway  Translocated | | LR axis formation  Import  Assembly  Secretion  Peroxisome biogenesis  Transfer  Secreted  PTS2-mediated import  Protein translocation  DNA |
| Molecular Function | | | |
| T4SS | | Near-Miss | General |
| 20 | | 13 | 4 |
| DNA-binding  DNA binding  Nucleotide binding  ATP-binding  ATP binding  ATPase activity  Single-stranded-DNA-binding  Rhicadhesin-binding  Mononucleotide binding  Kinase activity | | DNA binding  GTP-binding  ATP-binding  DNA-binding  ATP binding  PTPase activity  ATPase activity  GAP activity  Hydrolysis  SycE binding | Unfolding  Receptor binding  Pex7p binding  Nodal activity |

# Appendix F: Type IV Secretion System References

.

Abajy, M. Y., J. Kopec, et al. (2007). "A type IV-secretion-like system is required for conjugative DNA transport of broad-host-range plasmid pIP501 in gram-positive bacteria." J Bacteriol **189**(6): 2487-96.

Alegria, M. C., D. P. Souza, et al. (2005). "Identification of new protein-protein interactions involving the products of the chromosome- and plasmid-encoded type IV secretion loci of the phytopathogen *Xanthomonas axonopodis* pv. citri." J Bacteriol **187**(7): 2315-25.

Aly, K. A. and C. Baron (2007). "The VirB5 protein localizes to the T-pilus tips in *Agrobacterium tumefaciens*." Microbiology **153**(Pt 11): 3766-75.

Ammerman, N. C., M. S. Rahman, et al. (2008). "Characterization of Sec translocon-dependent extracytoplasmic proteins of *Rickettsia typhi*." J Bacteriol.

Anderson, L. B., A. V. Hertzel, et al. (1996). "*Agrobacterium tumefaciens* VirB7 and VirB9 form a disulfide-linked protein complex." Proc Natl Acad Sci U S A **93**(17): 8889-94.

Andersson, S. G. and C. G. Kurland (1995). "Genomic evolution drives the evolution of the translation system." Biochem Cell Biol **73**(11-12): 775-87.

Andersson, S. G., A. Zomorodipour, et al. (1998). "The genome sequence of *Rickettsia prowazekii* and the origin of mitochondria." Nature **396**(6707): 133-40.

Andersson, S. G., A. Zomorodipour, et al. (1995). "Unusual organization of the rRNA genes in *Rickettsia prowazekii*." J Bacteriol **177**(14): 4171-5.

Andrzejewska, J., S. K. Lee, et al. (2006). "Characterization of the pilin ortholog of the *Helicobacter pylori* type IV *cag* pathogenicity apparatus, a surface-associated protein expressed during infection." J Bacteriol **188**(16): 5865-77.

Angot, A., A. Vergunst, et al. (2007). "Exploitation of eukaryotic ubiquitin signaling pathways by effectors translocated by bacterial type III and type IV secretion systems." PLoS Pathog **3**(1): e3.

Arnold, K., L. Bordoli, et al. (2006). "The SWISS-MODEL workspace: a web-based environment for protein structure homology modelling." Bioinformatics **22**(2): 195-201.

Atmakuri, K., E. Cascales, et al. (2004). "Energetic components VirD4, VirB11 and VirB4 mediate early DNA transfer reactions required for bacterial type IV secretion." Mol Microbiol **54**(5): 1199-211.

Azad, A. F. (2007). "Pathogenic rickettsiae as bioterrorism agents." Clin Infect Dis **45 Suppl 1**: S52-5.

Azad, A. F. and C. B. Beard (1998). "Rickettsial pathogens and their arthropod vectors." Emerg Infect Dis **4**(2): 179-86.

Azad, A. F. and S. Radulovic (2003). "Pathogenic rickettsiae as bioterrorism agents." Ann N Y Acad Sci **990**: 734-8.

Backert, S., R. Fronzes, et al. (2008). "VirB2 and VirB5 proteins: specialized adhesins in bacterial type-IV secretion systems?" Trends Microbiol **16**(9): 409-13.

Bailey, S., D. Ward, et al. (2006). "*Agrobacterium tumefaciens* VirB8 structure reveals potential protein-protein interaction sites." Proc Natl Acad Sci U S A **103**(8): 2582-7.

Baldridge, G. D., N. Y. Burkhardt, et al. (2007). "Transposon insertion reveals pRM, a plasmid of *Rickettsia monacensis*." Appl Environ Microbiol **73**(15): 4984-95.

Baldridge, G. D., N. Y. Burkhardt, et al. (2008). "Plasmids of the pRM/pRF family occur in diverse *Rickettsia* species." Appl Environ Microbiol **74**(3): 645-52.

Bandyopadhyay, P., S. Liu, et al. (2007). "Environmental mimics and the Lvh type IVA secretion system contribute to virulence-related phenotypes of *Legionella pneumophila*." Infect Immun **75**(2): 723-35.

Baron, C. (2005). "From bioremediation to biowarfare: on the impact and mechanism of type IV secretion systems." FEMS Microbiol Lett **253**(2): 163-70.

Baron, C., O. C. D, et al. (2002). "Bacterial secrets of secretion: EuroConference on the biology of type IV secretion processes." Mol Microbiol **43**(5): 1359-65.

Baron, C., M. Llosa, et al. (1997). "VirB1, a component of the T-complex transfer machinery of *Agrobacterium tumefaciens*, is processed to a C-terminal secreted product, VirB1*." J Bacteriol **179**(4): 1203-10.

Baron, C., Y. R. Thorstenson, et al. (1997). "The lipoprotein VirB7 interacts with VirB9 in the membranes of *Agrobacterium tumefaciens*." J Bacteriol **179**(4): 1211-8.

Bayan, N., I. Guilvout, et al. (2006). "Secretins take shape." Mol Microbiol **60**(1): 1-4.

Bayer, M., R. Eferl, et al. (1995). "Gene 19 of plasmid R1 is required for both efficient conjugative DNA transfer and bacteriophage R17 infection." J Bacteriol **177**(15): 4279-88.

Bayer, M., R. Iberer, et al. (2001). "Functional and mutational analysis of p19, a DNA transfer protein with muramidase activity." J Bacteriol **183**(10): 3176-83.

Bayliss, R., R. Harris, et al. (2007). "NMR structure of a complex between the VirB9/VirB7 interaction domains of the pKM101 type IV secretion system." Proc Natl Acad Sci U S A **104**(5): 1673-8.

Beaupre, C. E., J. Bohne, et al. (1997). "Interactions between VirB9 and VirB10 membrane proteins involved in movement of DNA from *Agrobacterium tumefaciens* into plant cells." J Bacteriol **179**(1): 78-89.

Beijersbergen, A., S. J. Smith, et al. (1994). "Localization and topology of VirB proteins of *Agrobacterium tumefaciens*." Plasmid **32**(2): 212-8.

Bendtsen, J. D., H. Nielsen, et al. (2004). "Improved prediction of signal peptides: SignalP 3.0." J Mol Biol **340**(4): 783-95.

Berger, B. R. and P. J. Christie (1993). "The *Agrobacterium tumefaciens* *virB4* gene product is an essential virulence protein requiring an intact nucleoside triphosphate-binding domain." J Bacteriol **175**(6): 1723-34.

Berger, B. R. and P. J. Christie (1994). "Genetic complementation analysis of the *Agrobacterium tumefaciens* *virB* operon: *virB2* through *virB11* are essential virulence genes." J Bacteriol **176**(12): 3646-60.

Betzner, A. S. and W. Keck (1989). "Molecular cloning, overexpression and mapping of the *slt* gene encoding the soluble lytic transglycosylase of *Escherichia coli*." Mol Gen Genet **219**(3): 489-91.

Blackburn, N. T. and A. J. Clarke (2001). "Identification of four families of peptidoglycan lytic transglycosylases." J Mol Evol **52**(1): 78-84.

Blanc, G., H. Ogata, et al. (2007). "Lateral gene transfer between obligate intracellular bacteria: evidence from the *Rickettsia massiliae* genome." Genome Res **17**(11): 1657-64.

Bohne, J., A. Yim, et al. (1998). "The Ti plasmid increases the efficiency of *Agrobacterium tumefaciens* as a recipient in *virB*-mediated conjugal transfer of an IncQ plasmid." Proc Natl Acad Sci U S A **95**(12): 7057-62.

Boschiroli, M. L., S. Ouahrani-Bettache, et al. (2002). "Type IV secretion and *Brucella virulence*." Vet Microbiol **90**(1-4): 341-8.

Brown, M. R. and J. Barker (1999). "Unexplored reservoirs of pathogenic bacteria: protozoa and biofilms." Trends Microbiol **7**(1): 46-50.

Burns, D. L. (2003). "Type IV transporters of pathogenic bacteria." Curr Opin Microbiol **6**(1): 29-34.

Cao, T. B. and M. H. Saier, Jr. (2001). "Conjugal type IV macromolecular transfer systems of Gram-negative bacteria: organismal distribution, structural constraints and evolutionary conclusions." Microbiology **147**(Pt 12): 3201-14.

Carle, A., C. Hoppner, et al. (2006). "The *Brucella suis* type IV secretion system assembles in the cell envelope of the heterologous host *Agrobacterium tumefaciens* and increases IncQ plasmid pLS1 recipient competence." Infect Immun **74**(1): 108-17.

Cascales, E. and P. J. Christie (2003). "The versatile bacterial type IV secretion systems." Nat Rev Microbiol **1**(2): 137-49.

Cascales, E. and P. J. Christie (2004). "*Agrobacterium* VirB10, an ATP energy sensor required for type IV secretion." Proc Natl Acad Sci U S A **101**(49): 17228-33.

Cascales, E. and P. J. Christie (2004). "Definition of a bacterial type IV secretion pathway for a DNA substrate." Science **304**(5674): 1170-3.

Chao, C. C., D. Chelius, et al. (2004). "Proteome analysis of Madrid E strain of *Rickettsia* *prowazekii*." Proteomics **4**: 1280-92.

Chao, C. C., D. Chelius, et al. (2007). "Insight into the virulence of *Rickettsia prowazekii* by proteomic analysis and

comparison with an avirulent strain." Biochim Biophys Acta **1774**: 373-81.

Cheng, Z., X. Wang, et al. (2008). "Regulation of type IV secretion apparatus genes during *Ehrlichia chaffeensis* intracellular development by a previously unidentified protein." J Bacteriol **190**(6): 2096-105.

Cho, N. H., H. R. Kim, et al. (2007). "The *Orientia tsutsugamushi* genome reveals massive proliferation of conjugative type IV secretion system and host-cell interaction genes." Proc Natl Acad Sci U S A **104**(19): 7981-6.

Christie, P. J. (1997). "*Agrobacterium tumefaciens* T-complex transport apparatus: a paradigm for a new family of multifunctional transporters in eubacteria." J Bacteriol **179**(10): 3085-94.

Christie, P. J. (2001). "Type IV secretion: intercellular transfer of macromolecules by systems ancestrally related to conjugation machines." Mol Microbiol **40**(2): 294-305.

Christie, P. J. (2004). "Type IV secretion: the *Agrobacterium* VirB/D4 and related conjugation systems." Biochim Biophys Acta **1694**(1-3): 219-34.

Christie, P. J., K. Atmakuri, et al. (2005). "Biogenesis, architecture, and function of bacterial type IV secretion systems." Annu Rev Microbiol **59**: 451-85.

Christie, P. J. and E. Cascales (2005). "Structural and dynamic properties of bacterial type IV secretion systems (review)." Mol Membr Biol **22**(1-2): 51-61.

Christie, P. J. and J. P. Vogel (2000). "Bacterial type IV secretion: conjugation systems adapted to deliver effector molecules to host cells." Trends Microbiol **8**(8): 354-60.

Christie, P. J., J. E. Ward, Jr., et al. (1989). "A gene required for transfer of T-DNA to plants encodes an ATPase with autophosphorylating activity." Proc Natl Acad Sci U S A **86**(24): 9677-81.

Citovsky, V., A. Kapelnikov, et al. (2004). "Protein interactions involved in nuclear import of the *Agrobacterium* VirE2 protein *in vivo* and *in vitro*." J Biol Chem **279**(28): 29528-33.

Constantinesco, F., P. Forterre, et al. (2004). "A bipolar DNA helicase gene, *herA*, clusters with *rad50*, *mre11* and *nurA* genes in thermophilic archaea." Nucleic Acids Res **32**(4): 1439-47.

Covacci, A., J. L. Telford, et al. (1999). "*Helicobacter pylori* virulence and genetic geography." Science **284**(5418): 1328-33.

Cox, R., R. J. Mason-Gamer, et al. (2004). "Phylogenetic analysis of Sec7-domain-containing Arf nucleotide exchangers." Mol Biol Cell **15**(4): 1487-505.

Craik, D. J., N. L. Daly, et al. (2003). "Structures of naturally occurring circular proteins from bacteria." J Bacteriol **185**(14): 4011-21.

Crooks, G. E., G. Hon, et al. (2004). "WebLogo: a sequence logo generator." Genome Res **14**(6): 1188-90.

Dale, E. M., A. N. Binns, et al. (1993). "Construction and characterization of Tn*5virB*, a transposon that generates nonpolar mutations, and its use to define *virB8* as an essential virulence gene in *Agrobacterium tumefaciens*." J Bacteriol **175**(3): 887-91.

Dang, T. A. and P. J. Christie (1997). "The VirB4 ATPase of *Agrobacterium tumefaciens* is a cytoplasmic membrane protein exposed at the periplasmic surface." J Bacteriol **179**(2): 453-62.

Dang, T. A., X. R. Zhou, et al. (1999). "Dimerization of the *Agrobacterium tumefaciens* VirB4 ATPase and the effect of ATP-binding cassette mutations on the assembly and function of the T-DNA transporter." Mol Microbiol **32**(6): 1239-53.

Darby, A. C., N. H. Cho, et al. (2007). "Intracellular pathogens go extreme: genome evolution in the Rickettsiales." Trends Genet **23**(10): 511-20.

Darling, A. C., B. Mau, et al. (2004). "Mauve: multiple alignment of conserved genomic sequence with rearrangements." Genome Res **14**(7): 1394-403.

Das, A., L. B. Anderson, et al. (1997). "Delineation of the interaction domains of *Agrobacterium tumefaciens* VirB7 and VirB9 by use of the yeast two-hybrid assay." J Bacteriol **179**(11): 3404-9.

Das, A. and Y. H. Xie (1998). "Construction of transposon Tn*3phoA*: its application in defining the membrane topology of the *Agrobacterium tumefaciens* DNA transfer proteins." Mol Microbiol **27**(2): 405-14.

Das, A. and Y. H. Xie (2000). "The *Agrobacterium* T-DNA transport pore proteins VirB8, VirB9, and VirB10 interact with one another." J Bacteriol **182**(3): 758-63.

De Buck, E., J. Anne, et al. (2007). "The role of protein secretion systems in the virulence of the intracellular pathogen *Legionella pneumophila*." Microbiology **153**(Pt 12): 3948-53.

de Paz, H. D., F. J. Sangari, et al. (2005). "Functional interactions between type IV secretion systems involved in DNA transfer and virulence." Microbiology **151**(Pt 11): 3505-16.

Dehio, C. (2008). "Infection-associated type IV secretion systems of *Bartonella* and their diverse roles in host cell interaction." Cell Microbiol.

den Hartigh, A. B., H. G. Rolan, et al. (2008). "VirB3 to VirB6 and VirB8 to VirB11, but not VirB7, are essential for mediating persistence of *Brucella* in the reticuloendothelial system." J Bacteriol **190**(13): 4427-36.

den Hartigh, A. B., Y. H. Sun, et al. (2004). "Differential requirements for VirB1 and VirB2 during *Brucella abortus* infection." Infect Immun **72**(9): 5143-9.

Dijkstra, A. J. and W. Keck (1996). "Peptidoglycan as a barrier to transenvelope transport." J Bacteriol **178**(19): 5555-62.

Dozot, M., R. A. Boigegrain, et al. (2006). "The stringent response mediator Rsh is required for *Brucella melitensis* and *Brucella suis* virulence, and for expression of the type IV secretion system *virB*." Cell Microbiol **8**(11): 1791-802.

Draper, O., R. Middleton, et al. (2006). "Topology of the VirB4 C terminus in the *Agrobacterium tumefaciens* VirB/D4 type IV secretion system." J Biol Chem **281**(49): 37628-35.

Draskovic, I. and D. Dubnau (2005). "Biogenesis of a putative channel protein, ComEC, required for DNA uptake: membrane topology, oligomerization and formation of disulphide bonds." Mol Microbiol **55**(3): 881-96.

Dreher-Lesnick, S. M., S. M. Ceraul, et al. (2008). "Genome-wide screen for temperature-regulated genes of the obligate intracellular bacterium, *Rickettsia typhi*." BMC Microbiol **8**: 61.

Duckely, M. and B. Hohn (2003). "The VirE2 protein of *Agrobacterium tumefaciens*: the Yin and Yang of T-DNA transfer." FEMS Microbiol Lett **223**(1): 1-6.

Edgar, R. C. (2004). "MUSCLE: a multiple sequence alignment method with reduced time and space complexity." BMC Bioinformatics **5**: 113.

Edgar, R. C. (2004). "MUSCLE: multiple sequence alignment with high accuracy and high throughput." Nucleic Acids Res **32**(5): 1792-7.

Eisenbrandt, R., M. Kalkum, et al. (1999). "Conjugative pili of IncP plasmids, and the Ti plasmid T pilus are composed of cyclic subunits." J Biol Chem **274**(32): 22548-55.

Eisenbrandt, R., M. Kalkum, et al. (2000). "Maturation of IncP pilin precursors resembles the catalytic Dyad-like mechanism of leader peptidases." J Bacteriol **182**(23): 6751-61.

Ellison, D. W., T. R. Clark, et al. (2008). "Genomic comparison of virulent *Rickettsia rickettsii* Sheila Smith and avirulent *Rickettsia rickettsii* Iowa." Infect Immun **76**(2): 542-50.

Eremeeva, M. E., A. Madan, et al. (2005). "New perspectives on rickettsial evolution from new genome sequences of *rickettsia*, particularly *R*. *canadensis*, and *Orientia* *tsutsugamushi*." Ann N Y Acad Sci **1063**: 47-63.

Escobar, M. A. and A. M. Dandekar (2003). "*Agrobacterium tumefaciens* as an agent of disease." Trends Plant Sci **8**(8): 380-6.

Fernandez, D., T. A. Dang, et al. (1996). "The *Agrobacterium tumefaciens* *virB7* gene product, a proposed component of the T-complex transport apparatus, is a membrane-associated lipoprotein exposed at the periplasmic surface." J Bacteriol **178**(11): 3156-67.

Fernandez, D., G. M. Spudich, et al. (1996). "The *Agrobacterium tumefaciens* VirB7 lipoprotein is required for stabilization of VirB proteins during assembly of the T-complex transport apparatus." J Bacteriol **178**(11): 3168-76.

Finberg, K. E., T. R. Muth, et al. (1995). "Interactions of VirB9, -10, and -11 with the membrane fraction of *Agrobacterium tumefaciens*: solubility studies provide evidence for tight associations." J Bacteriol **177**(17): 4881-9.

Fischer, W., J. Puls, et al. (2001). "Systematic mutagenesis of the *Helicobacter pylori cag* pathogenicity island: essential genes for CagA translocation in host cells and induction of interleukin-8." Mol Microbiol **42**(5): 1337-48.

Fritsche, T. R., R. K. Gautom, et al. (1993). "Occurrence of bacterial endosymbionts in *Acanthamoeba* spp. isolated from corneal and environmental specimens and contact lenses." J Clin Microbiol **31**(5): 1122-6.

Fullner, K. J. (1998). "Role of *Agrobacterium virB* genes in transfer of T complexes and RSF1010." J Bacteriol **180**(2): 430-4.

Fullner, K. J., K. M. Stephens, et al. (1994). "An essential virulence protein of *Agrobacterium tumefaciens*, VirB4, requires an intact mononucleotide binding domain to function in transfer of T-DNA." Mol Gen Genet **245**(6): 704-15.

Fuxelius, H. H., A. Darby, et al. (2007). "The genomic and metabolic diversity of *Rickettsia*." Res Microbiol **158**(10): 745-53.

Fuxelius, H. H., A. C. Darby, et al. (2008). "Visualization of pseudogenes in intracellular bacteria reveals the different tracks to gene destruction." Genome Biol **9**(2): R42.

Ge, H., Y. Y. Chuang, et al. (2004). "Comparative genomics of *Rickettsia prowazekii* Madrid E and Breinl strains." J Bacteriol **186**(2): 556-65.

Gillespie, J. J., M. S. Beier, et al. (2007). "Plasmids and rickettsial evolution: insight from *Rickettsia felis*." PLoS ONE **2**(3): e266.

Gillespie, J. J., K. Williams, et al. (2008). "Rickettsia phylogenomics: unwinding the intricacies of obligate intracellular life." PLoS ONE **3**(4): e2018.

Gilmour, M. W., J. E. Gunton, et al. (2003). "Interaction between the IncHI1 plasmid R27 coupling protein and type IV secretion system: TraG associates with the coiled-coil mating pair formation protein TrhB." Mol Microbiol **49**(1): 105-16.

Gomis-Ruth, F. X. and M. Coll (2001). "Structure of TrwB, a gatekeeper in bacterial conjugation." Int J Biochem Cell Biol **33**(9): 839-43.

Gomis-Ruth, F. X., F. de la Cruz, et al. (2002). "Structure and role of coupling proteins in conjugal DNA transfer." Res Microbiol **153**(4): 199-204.

Gomis-Ruth, F. X., G. Moncalian, et al. (2002). "Conjugative plasmid protein TrwB, an integral membrane type IV secretion system coupling protein. Detailed structural features and mapping of the active site cleft." J Biol Chem **277**(9): 7556-66.

Gomis-Ruth, F. X., G. Moncalian, et al. (2001). "The bacterial conjugation protein TrwB resembles ring helicases and F1-ATPase." Nature **409**(6820): 637-41.

Gray, M. W., G. Burger, et al. (1999). "Mitochondrial evolution." Science **283**(5407): 1476-81.

Gunton, J. E., M. W. Gilmour, et al. (2005). "Subcellular localization and functional domains of the coupling protein, TraG, from IncHI1 plasmid R27." Microbiology **151**(Pt 11): 3549-61.

Guyon, P., M. D. Chilton, et al. (1980). "Agropine in "null-type" crown gall tumors: Evidence for generality of the opine concept." Proc Natl Acad Sci U S A **77**(5): 2693-2697.

Haft, R. J., E. G. Gachelet, et al. (2007). "*In vivo* oligomerization of the F conjugative coupling protein TraD." J Bacteriol **189**(18): 6626-34.

Hall, J. and H. Voelz (1985). "Bacterial endosymbionts of *Acanthamoeba* sp." J Parasitol **71**(1): 89-95.

Hamilton, C. M., H. Lee, et al. (2000). "TraG from RP4 and TraG and VirD4 from Ti plasmids confer relaxosome specificity to the conjugal transfer system of pTiC58." J Bacteriol **182**(6): 1541-8.

Hapfelmeier, S., N. Domke, et al. (2000). "VirB6 is required for stabilization of VirB5 and VirB3 and formation of VirB7 homodimers in *Agrobacterium tumefaciens*." J Bacteriol **182**(16): 4505-11.

Hare, S., R. Bayliss, et al. (2006). "A large domain swap in the VirB11 ATPase of *Brucella suis* leaves the hexameric assembly intact." J Mol Biol **360**(1): 56-66.

Hare, S., W. Fischer, et al. (2007). "Identification, structure and mode of action of a new regulator of the *Helicobacter pylori* HP0525 ATPase." Embo J **26**(23): 4926-34.

Harris, R. L., V. Hombs, et al. (2001). "Evidence that F-plasmid proteins TraV, TraK and TraB assemble into an envelope-spanning structure in *Escherichia coli*." Mol Microbiol **42**(3): 757-66.

Hilleringmann, M., W. Pansegrau, et al. (2006). "Inhibitors of *Helicobacter pylori* ATPase Cagalpha block CagA transport and *cag* virulence." Microbiology **152**(Pt 10): 2919-30.

Hobbs, M. and J. S. Mattick (1993). "Common components in the assembly of type 4 fimbriae, DNA transfer systems, filamentous phage and protein-secretion apparatus: a general system for the formation of surface-associated protein complexes." Mol Microbiol **10**(2): 233-43.

Hofreuter, D., S. Odenbreit, et al. (2001). "Natural transformation competence in *Helicobacter pylori* is mediated by the basic components of a type IV secretion system." Mol Microbiol **41**(2): 379-91.

Hoppner, C., A. Carle, et al. (2005). "The putative lytic transglycosylase VirB1 from *Brucella suis* interacts with the type IV secretion system core components VirB8, VirB9 and VirB11." Microbiology **151**(Pt 11): 3469-82.

Hormaeche, I., I. Alkorta, et al. (2002). "Purification and properties of TrwB, a hexameric, ATP-binding integral membrane protein essential for R388 plasmid conjugation." J Biol Chem **277**(48): 46456-62.

Horn, M., T. R. Fritsche, et al. (1999). "Novel bacterial endosymbionts of *Acanthamoeba* spp. related to the *Paramecium caudatum* symbiont *Caedibacter caryophilus*." Environ Microbiol **1**(4): 357-67.

Huelsenbeck, J. P. and F. Ronquist (2001). "MRBAYES: Bayesian inference of phylogenetic trees." Bioinformatics **17**(8): 754-5.

Jakubowski, S. J., E. Cascales, et al. (2005). "*Agrobacterium tumefaciens* VirB9, an outer-membrane-associated component of a type IV secretion system, regulates substrate selection and T-pilus biogenesis." J Bacteriol **187**(10): 3486-95.

Jakubowski, S. J., V. Krishnamoorthy, et al. (2004). "*Agrobacterium tumefaciens* VirB6 domains direct the ordered export of a DNA substrate through a type IV secretion system." J Mol Biol **341**(4): 961-77.

Jakubowski, S. J., V. Krishnamoorthy, et al. (2003). "*Agrobacterium tumefaciens* VirB6 protein participates in formation of VirB7 and VirB9 complexes required for type IV secretion." J Bacteriol **185**(9): 2867-78.

Jones, A. L., E. M. Lai, et al. (1996). "VirB2 is a processed pilin-like protein encoded by the *Agrobacterium tumefaciens* Ti plasmid." J Bacteriol **178**(19): 5706-11.

Jones, A. L., K. Shirasu, et al. (1994). "The product of the *virB4* gene of *Agrobacterium tumefaciens* promotes accumulation of VirB3 protein." J Bacteriol **176**(17): 5255-61.

Judd, P. K., R. B. Kumar, et al. (2005). "Spatial location and requirements for the assembly of the *Agrobacterium tumefaciens* type IV secretion apparatus." Proc Natl Acad Sci U S A **102**(32): 11498-503.

Judd, P. K., R. B. Kumar, et al. (2005). "The type IV secretion apparatus protein VirB6 of *Agrobacterium tumefaciens* localizes to a cell pole." Mol Microbiol **55**(1): 115-24.

Judd, P. K., D. Mahli, et al. (2005). "Molecular characterization of the *Agrobacterium tumefaciens* DNA transfer protein VirB6." Microbiology **151**(Pt 11): 3483-92.

Juncker, A. S., H. Willenbrock, et al. (2003). "Prediction of lipoprotein signal peptides in Gram-negative bacteria." Protein Sci **12**(8): 1652-62.

Kado, C. I. (1994). "Promiscuous DNA transfer system of *Agrobacterium tumefaciens*: role of the *virB* operon in sex pilus assembly and synthesis." Mol Microbiol **12**(1): 17-22.

Kalkum, M., R. Eisenbrandt, et al. (2004). "Protein circlets as sex pilus subunits." Curr Protein Pept Sci **5**(5): 417-24.

Karnholz, A., C. Hoefler, et al. (2006). "Functional and topological characterization of novel components of the *comB* DNA transformation competence system in *Helicobacter pylori*." J Bacteriol **188**(3): 882-93.

Koch, A. L. (1990). "The surface stress theory for the case of *Escherichia coli*: the paradoxes of gram-negative growth." Res Microbiol **141**(1): 119-30.

Koonin, E. V. and K. E. Rudd (1994). "A conserved domain in putative bacterial and bacteriophage transglycosylases." Trends Biochem Sci **19**(3): 106-7.

Koraimann, G. (2003). "Lytic transglycosylases in macromolecular transport systems of Gram-negative bacteria." Cell Mol Life Sci **60**(11): 2371-88.

Krall, L., U. Wiedemann, et al. (2002). "Detergent extraction identifies different VirB protein subassemblies of the type IV secretion machinery in the membranes of *Agrobacterium tumefaciens*." Proc Natl Acad Sci U S A **99**(17): 11405-10.

Krause, S., M. Barcena, et al. (2000). "Sequence-related protein export NTPases encoded by the conjugative transfer region of RP4 and by the *cag* pathogenicity island of *Helicobacter pylori* share similar hexameric ring structures." Proc Natl Acad Sci U S A **97**(7): 3067-72.

Krause, S., W. Pansegrau, et al. (2000). "Enzymology of type IV macromolecule secretion systems: the conjugative transfer regions of plasmids RP4 and R388 and the *cag* pathogenicity island of *Helicobacter pylori* encode structurally and functionally related nucleoside triphosphate hydrolases." J Bacteriol **182**(10): 2761-70.

Krogh, A., B. Larsson, et al. (2001). "Predicting transmembrane protein topology with a hidden Markov model: application to complete genomes." J Mol Biol **305**(3): 567-80.

Kuldau, G. A., G. De Vos, et al. (1990). "The *virB* operon of *Agrobacterium tumefaciens* pTiC58 encodes 11 open reading frames." Mol Gen Genet **221**(2): 256-66.

Kumar, R. B. and A. Das (2001). "Functional analysis of the *Agrobacterium tumefaciens* T-DNA transport pore protein VirB8." J Bacteriol **183**(12): 3636-41.

Kumar, R. B., Y. H. Xie, et al. (2000). "Subcellular localization of the *Agrobacterium tumefaciens* T-DNA transport pore proteins: VirB8 is essential for the assembly of the transport pore." Mol Microbiol **36**(3): 608-17.

Kurbanova, I. V., V. A. Velikov, et al. (2001). "Expression of *virB2* protein-containing structures on *Agrobacterium* in mating cultures." Antonie Van Leeuwenhoek **79**(3-4): 291-5.

Kutter, S., R. Buhrdorf, et al. (2008). "Protein subassemblies of the *Helicobacter pylori* Cag type IV secretion system revealed by localization and interaction studies." J Bacteriol **190**(6): 2161-71.

La, M. V., P. Francois, et al. (2007). "Development of a method for recovering rickettsial RNA from infected cells to analyze gene expression profiling of obligate intracellular bacteria." J Microbiol Methods **71**(3): 292-7.

Lai, E. M., O. Chesnokova, et al. (2000). "Genetic and environmental factors affecting T-pilin export and T-pilus biogenesis in relation to flagellation of *Agrobacterium tumefaciens*." J Bacteriol **182**(13): 3705-16.

Lai, E. M., R. Eisenbrandt, et al. (2002). "Biogenesis of T pili in *Agrobacterium tumefaciens* requires precise VirB2 propilin cleavage and cyclization." J Bacteriol **184**(1): 327-30.

Lai, E. M. and C. I. Kado (1998). "Processed VirB2 is the major subunit of the promiscuous pilus of *Agrobacterium tumefaciens*." J Bacteriol **180**(10): 2711-7.

Lai, E. M. and C. I. Kado (2000). "The T-pilus of *Agrobacterium tumefaciens*." Trends Microbiol **8**(8): 361-9.

Lanka, E. and B. M. Wilkins (1995). "DNA processing reactions in bacterial conjugation." Annu Rev Biochem **64**: 141-69.

Lawley, T. D., W. A. Klimke, et al. (2003). "F factor conjugation is a true type IV secretion system." FEMS Microbiol Lett **224**(1): 1-15.

Lehnherr, H., A. M. Hansen, et al. (1998). "Penetration of the bacterial cell wall: a family of lytic transglycosylases in bacteriophages and conjugative plasmids." Mol Microbiol **30**(2): 454-7.

Lessl, M. and E. Lanka (1994). "Common mechanisms in bacterial conjugation and Ti-mediated T-DNA transfer to plant cells." Cell **77**(3): 321-4.

Lessl, M., W. Pansegrau, et al. (1992). "Relationship of DNA-transfer-systems: essential transfer factors of plasmids RP4, Ti and F share common sequences." Nucleic Acids Res **20**(22): 6099-100.

Lin, T. S. and C. I. Kado (1993). "The *virD4* gene is required for virulence while *virD3* and orf5 are not required for virulence of *Agrobacterium tumefaciens*." Mol Microbiol **9**(4): 803-12.

Liu, Z. and A. N. Binns (2003). "Functional subsets of the *virB* type IV transport complex proteins involved in the capacity of *Agrobacterium tumefaciens* to serve as a recipient in *virB*-mediated conjugal transfer of plasmid RSF1010." J Bacteriol **185**(11): 3259-69.

Llosa, M., S. Zunzunegui, et al. (2003). "Conjugative coupling proteins interact with cognate and heterologous VirB10-like proteins while exhibiting specificity for cognate relaxosomes." Proc Natl Acad Sci U S A **100**(18): 10465-70.

Llosa, M., J. Zupan, et al. (2000). "The N- and C-terminal portions of the *Agrobacterium* VirB1 protein independently enhance tumorigenesis." J Bacteriol **182**(12): 3437-45.

Lu, J. and L. S. Frost (2005). "Mutations in the C-terminal region of TraM provide evidence for *in vivo* TraM-TraD interactions during F-plasmid conjugation." J Bacteriol **187**(14): 4767-73.

Machon, C., S. Rivas, et al. (2002). "TrwD, the hexameric traffic ATPase encoded by plasmid R388, induces membrane destabilization and hemifusion of lipid vesicles." J Bacteriol **184**(6): 1661-8.

Maher, D., R. Sherburne, et al. (1993). "H-pilus assembly kinetics determined by electron microscopy." J Bacteriol **175**(8): 2175-83.

Malek, J. A., J. M. Wierzbowski, et al. (2004). "Protein interaction mapping on a functional shotgun sequence of *Rickettsia sibirica*." Nucleic Acids Res **32**(3): 1059-64.

Martinez, J. J., S. Seveau, et al. (2005). "Ku70, a component of DNA-dependent protein kinase, is a mammalian receptor for *Rickettsia conorii*." Cell **123**(6): 1013-23.

Masui, S., T. Sasaki, et al. (2000). "Genes for the type IV secretion system in an intracellular symbiont, *Wolbachia*, a causative agent of various sexual alterations in arthropods." J Bacteriol **182**(22): 6529-31.

McLeod, M. P., X. Qin, et al. (2004). "Complete genome sequence of *Rickettsia typhi* and comparison with sequences of other rickettsiae." J Bacteriol **186**(17): 5842-55.

Medini, D., A. Covacci, et al. (2006). "Protein homology network families reveal step-wise diversification of Type III and Type IV secretion systems." PLoS Comput Biol **2**(12): e173.

Metzger, S., E. Sarubbi, et al. (1989). "Protein sequences encoded by the *relA* and the *spoT* genes of *Escherichia coli* are interrelated." J Biol Chem **264**(16): 9122-5.

Middleton, R., K. Sjolander, et al. (2005). "Predicted hexameric structure of the *Agrobacterium* VirB4 C terminus suggests VirB4 acts as a docking site during type IV secretion." Proc Natl Acad Sci U S A **102**(5): 1685-90.

Moncalian, G., E. Cabezon, et al. (1999). "Characterization of ATP and DNA binding activities of TrwB, the coupling protein essential in plasmid R388 conjugation." J Biol Chem **274**(51): 36117-24.

Motallebi-Veshareh, M., D. Balzer, et al. (1992). "Conjugative transfer functions of broad-host-range plasmid RK2 are coregulated with vegetative replication." Mol Microbiol **6**(7): 907-20.

Mushegian, A. R., K. J. Fullner, et al. (1996). "A family of lysozyme-like virulence factors in bacterial pathogens of plants and animals." Proc Natl Acad Sci U S A **93**(14): 7321-6.

Nagai, H., J. C. Kagan, et al. (2002). "A bacterial guanine nucleotide exchange factor activates ARF on *Legionella phagosomes*." Science **295**(5555): 679-82.

Niu, H., Y. Rikihisa, et al. (2006). "Differential expression of VirB9 and VirB6 during the life cycle of *Anaplasma phagocytophilum* in human leucocytes is associated with differential binding and avoidance of lysosome pathway." Cell Microbiol **8**(3): 523-34.

Nogami, S., S. Satoh, et al. (2003). "Taxilin; a novel syntaxin-binding protein that is involved in Ca2+-dependent exocytosis in neuroendocrine cells." Genes Cells **8**(1): 17-28.

Norris, V. and B. Manners (1993). "Deformations in the cytoplasmic membrane of *Escherichia coli* direct the synthesis of peptidoglycan. The hernia model." Biophys J **64**(6): 1691-700.

Nystedt, B., A. C. Frank, et al. (2008). "Diversifying selection and concerted evolution of a type IV secretion system in *Bartonella*." Mol Biol Evol **25**(2): 287-300.

Ogata, H., S. Audic, et al. (2000). "Selfish DNA in protein-coding genes of *Rickettsia*." Science **290**(5490): 347-50.

Ogata, H., S. Audic, et al. (2001). "Mechanisms of evolution in *Rickettsia conorii* and *R*. *prowazekii*." Science **293**(5537): 2093-8.

Ogata, H., B. La Scola, et al. (2006). "Genome sequence of *Rickettsia bellii* illuminates the role of amoebae in gene exchanges between intracellular pathogens." PLoS Genet **2**(5): e76.

Ogata, H., P. Renesto, et al. (2005). "The genome sequence of *Rickettsia felis* identifies the first putative conjugative plasmid in an obligate intracellular parasite." PLoS Biol **3**(8): e248.

Ogawa, M., P. Renesto, et al. (2007). "Proteome analysis of *Rickettsia felis* highlights the expression profile of intracellular bacteria." Proteomics **7**(8): 1232-48.

Ohashi, N., N. Zhi, et al. (2002). "Characterization and transcriptional analysis of gene clusters for a type IV secretion machinery in human granulocytic and monocytic ehrlichiosis agents." Infect Immun **70**(4): 2128-38.

Okamoto, S., A. Toyoda-Yamamoto, et al. (1991). "Localization and orientation of the VirD4 protein of *Agrobacterium tumefaciens* in the cell membrane." Mol Gen Genet **228**(1-2): 24-32.

Olsen, G. J., C. R. Woese, et al. (1994). "The winds of (evolutionary) change: breathing new life into microbiology." J Bacteriol **176**(1): 1-6.

Pansegrau, W. and E. Lanka (1996). "Enzymology of DNA transfer by conjugative mechanisms." Prog Nucleic Acid Res Mol Biol **54**: 197-251.

Paschos, A., G. Patey, et al. (2006). "Dimerization and interactions of *Brucella suis* VirB8 with VirB4 and VirB10 are required for its biological activity." Proc Natl Acad Sci U S A **103**(19): 7252-7.

Patey, G., Z. Qi, et al. (2006). "Swapping of periplasmic domains between *Brucella suis* VirB8 and a pSB102 VirB8 homologue allows heterologous complementation." Infect Immun **74**(8): 4945-9.

Peters, J., D. P. Wilson, et al. (2007). "Type III secretion a la *Chlamydia*." Trends Microbiol **15**(6): 241-51.

Planet, P. J., S. C. Kachlany, et al. (2001). "Phylogeny of genes for secretion NTPases: identification of the widespread *tadA* subfamily and development of a diagnostic key for gene classification." Proc Natl Acad Sci U S A **98**(5): 2503-8.

Posada, D. and K. A. Crandall (1998). "MODELTEST: testing the model of DNA substitution." Bioinformatics **14**(9): 817-8.

Postle, K. and R. J. Kadner (2003). "Touch and go: tying TonB to transport." Mol Microbiol **49**(4): 869-82.

Pugsley, A. P. (1993). "The complete general secretory pathway in gram-negative bacteria." Microbiol Rev **57**(1): 50-108.

Rambaut, A. and A. J. Drummond (2007). Tracer v1.4, Available from <http://beast.bio.ed.ac.uk/Tracer.>

Rances, E., D. Voronin, et al. (2008). "Genetic and functional characterization of the type IV secretion system in *Wolbachia*." J Bacteriol.

Rashkova, S., G. M. Spudich, et al. (1997). "Characterization of membrane and protein interaction determinants of the *Agrobacterium tumefaciens* VirB11 ATPase." J Bacteriol **179**(3): 583-91.

Rivas, S., S. Bolland, et al. (1997). "TrwD, a protein encoded by the IncW plasmid R388, displays an ATP hydrolase activity essential for bacterial conjugation." J Biol Chem **272**(41): 25583-90.

Robertus, J. D., A. F. Monzingo, et al. (1998). "Structural analysis shows five glycohydrolase families diverged from a common ancestor." J Exp Zool **282**(1-2): 127-32.

Rohde, M., J. Puls, et al. (2003). "A novel sheathed surface organelle of the *Helicobacter pylori cag* type IV secretion system." Mol Microbiol **49**(1): 219-34.

Ronquist, F. and J. P. Huelsenbeck (2003). "MrBayes 3: Bayesian phylogenetic inference under mixed models." Bioinformatics **19**(12): 1572-4.

Rowbotham, T. J. (1980). "Preliminary report on the pathogenicity of *Legionella pneumophila* for freshwater and soil amoebae." J Clin Pathol **33**(12): 1179-83.

Roy, C. R. and L. G. Tilney (2002). "The road less traveled: transport of *Legionella* to the endoplasmic reticulum." J Cell Biol **158**(3): 415-9.

Saenz, H. L., P. Engel, et al. (2007). "Genomic analysis of *Bartonella* identifies type IV secretion systems as host adaptability factors." Nat Genet **39**(12): 1469-76.

Sagulenko, V., E. Sagulenko, et al. (2001). "VirB7 lipoprotein is exocellular and associates with the *Agrobacterium tumefaciens* T pilus." J Bacteriol **183**(12): 3642-51.

Salmond, G. (1994). "Secretion of Extracellular Virulence Factors by Plant Pathogenic Bacteria." Annual Review of Phytopathology **32**: 181-200.

Sastre, J. I., E. Cabezon, et al. (1998). "The carboxyl terminus of protein TraD adds specificity and efficiency to F-plasmid conjugative transfer." J Bacteriol **180**(22): 6039-42.

Savvides, S. N., H. J. Yeo, et al. (2003). "VirB11 ATPases are dynamic hexameric assemblies: new insights into bacterial type IV secretion." Embo J **22**(9): 1969-80.

Schmidt-Eisenlohr, H., N. Domke, et al. (1999). "Vir proteins stabilize VirB5 and mediate its association with the T pilus of *Agrobacterium tumefaciens*." J Bacteriol **181**(24): 7485-92.

Schmidt-Eisenlohr, H., N. Domke, et al. (1999). "TraC of IncN plasmid pKM101 associates with membranes and extracellular high-molecular-weight structures in *Escherichia coli*." J Bacteriol **181**(18): 5563-71.

Schneider, T. D. and R. M. Stephens (1990). "Sequence logos: a new way to display consensus sequences." Nucleic Acids Res **18**(20): 6097-100.

Schroder, G., S. Krause, et al. (2002). "TraG-like proteins of DNA transfer systems and of the *Helicobacter pylori* type IV secretion system: inner membrane gate for exported substrates?" J Bacteriol **184**(10): 2767-79.

Schroder, G. and E. Lanka (2003). "TraG-like proteins of type IV secretion systems: functional dissection of the multiple activities of TraG (RP4) and TrwB (R388)." J Bacteriol **185**(15): 4371-81.

Schulein, R. and C. Dehio (2002). "The VirB/VirD4 type IV secretion system of *Bartonella* is essential for establishing intraerythrocytic infection." Mol Microbiol **46**(4): 1053-67.

Schulein, R., P. Guye, et al. (2005). "A bipartite signal mediates the transfer of type IV secretion substrates of *Bartonella henselae* into human cells." Proc Natl Acad Sci U S A **102**(3): 856-61.

Segal, E. D., J. Cha, et al. (1999). "Altered states: involvement of phosphorylated CagA in the induction of host cellular growth changes by *Helicobacter pylori*." Proc Natl Acad Sci U S A **96**(25): 14559-64.

Segal, G., J. J. Russo, et al. (1999). "Relationships between a new type IV secretion system and the icm/dot virulence system of *Legionella pneumophila*." Mol Microbiol **34**(4): 799-809.

Segal, G. and H. A. Shuman (1998). "How is the intracellular fate of the *Legionella pneumophila* phagosome determined?" Trends Microbiol **6**(7): 253-5.

Sexton, J. A. and J. P. Vogel (2002). "Type IVB secretion by intracellular pathogens." Traffic **3**(3): 178-85.

Shamaei-Tousi, A., R. Cahill, et al. (2004). "Interaction between protein subunits of the type IV secretion system of *Bartonella henselae*." J Bacteriol **186**(14): 4796-801.

Shirasu, K. and C. I. Kado (1993). "Membrane location of the Ti plasmid VirB proteins involved in the biosynthesis of a pilin-like conjugative structure on *Agrobacterium tumefaciens*." FEMS Microbiol Lett **111**(2-3): 287-94.

Shirasu, K., P. Morel, et al. (1990). "Characterization of the *virB* operon of an *Agrobacterium tumefaciens* Ti plasmid: nucleotide sequence and protein analysis." Mol Microbiol **4**(7): 1153-63.

Snyder, E. E., N. Kampanya, et al. (2007). "PATRIC: the VBI PathoSystems Resource Integration Center." Nucleic Acids Res **35**(Database issue): D401-6.

Spudich, G. M., D. Fernandez, et al. (1996). "Intermolecular disulfide bonds stabilize VirB7 homodimers and VirB7/VirB9 heterodimers during biogenesis of the *Agrobacterium tumefaciens* T-complex transport apparatus." Proc Natl Acad Sci U S A **93**(15): 7512-7.

Stachel, S. E. and P. C. Zambryski (1986). "*virA* and *virG* control the plant-induced activation of the T-DNA transfer process of *A*. *tumefaciens*." Cell **46**(3): 325-33.

Stephens, K. M., C. Roush, et al. (1995). "*Agrobacterium tumefaciens* VirB11 protein requires a consensus nucleotide-binding site for function in virulence." J Bacteriol **177**(1): 27-36.

Stern, A., A. Doron-Faigenboim, et al. (2007). "Selecton 2007: advanced models for detecting positive and purifying selection using a Bayesian inference approach." Nucleic Acids Res **35**(Web Server issue): W506-11.

Stothard, D. R. and P. A. Fuerst (1995). "Evolutionary analysis of the spotted fever and thyphus groups of *Rickettsia* using 16S rRNA gene sequences." Syst Appl Microbiol **18**: 52-61.

Tanaka, J., T. Suzuki, et al. (2003). "Structural definition on the surface of *Helicobacter pylori* type IV secretion apparatus." Cell Microbiol **5**(6): 395-404.

Tato, I., I. Matilla, et al. (2007). "The ATPase activity of the DNA transporter TrwB is modulated by protein TrwA: implications for a common assembly mechanism of DNA translocating motors." J Biol Chem **282**(35): 25569-76.

Tato, I., S. Zunzunegui, et al. (2005). "TrwB, the coupling protein involved in DNA transport during bacterial conjugation, is a DNA-dependent ATPase." Proc Natl Acad Sci U S A **102**(23): 8156-61.

Terradot, L., R. Bayliss, et al. (2005). "Structures of two core subunits of the bacterial type IV secretion system, VirB8 from *Brucella suis* and ComB10 from *Helicobacter pylori*." Proc Natl Acad Sci U S A **102**(12): 4596-601.

Thompson, D. V., L. S. Melchers, et al. (1988). "Analysis of the complete nucleotide sequence of the *Agrobacterium tumefaciens* *virB* operon." Nucleic Acids Res **16**(10): 4621-36.

Thorstenson, Y. R. and P. C. Zambryski (1994). "The essential virulence protein VirB8 localizes to the inner membrane of *Agrobacterium tumefaciens*." J Bacteriol **176**(6): 1711-7.

Tyson, G. W., J. Chapman, et al. (2004). "Community structure and metabolism through reconstruction of microbial genomes from the environment." Nature **428**(6978): 37-43.

Tzfira, T. and V. Citovsky (2002). "Partners-in-infection: host proteins involved in the transformation of plant cells by *Agrobacterium*." Trends Cell Biol **12**(3): 121-9.

Tzfira, T., M. Vaidya, et al. (2004). "Involvement of targeted proteolysis in plant genetic transformation by *Agrobacterium*." Nature **431**(7004): 87-92.

van Asselt, E. J., A. M. Thunnissen, et al. (1999). "High resolution crystal structures of the *Escherichia coli* lytic transglycosylase Slt70 and its complex with a peptidoglycan fragment." J Mol Biol **291**(4): 877-98.

Vergunst, A. C., M. C. van Lier, et al. (2005). "Positive charge is an important feature of the C-terminal transport signal of the VirB/D4-translocated proteins of *Agrobacterium*." Proc Natl Acad Sci U S A **102**(3): 832-7.

Verma, A. and D. L. Burns (2007). "Requirements for assembly of PtlH with the pertussis toxin transporter apparatus of *Bordetella pertussis*." Infect Immun **75**(5): 2297-306.

Vogel, J. P. and R. R. Isberg (1999). "Cell biology of *Legionella pneumophila*." Curr Opin Microbiol **2**(1): 30-4.

Vollmer, W. and J. V. Holtje (2001). "Morphogenesis of *Escherichia coli*." Curr Opin Microbiol **4**(6): 625-33.

Walker, D. H. and N. Ismail (2008). "Emerging and re-emerging rickettsioses: endothelial cell infection and early disease events." Nat Rev Microbiol **6**(5): 375-86.

Ward, D. V., O. Draper, et al. (2002). "Peptide linkage mapping of the *Agrobacterium tumefaciens vir*-encoded type IV secretion system reveals protein subassemblies." Proc Natl Acad Sci U S A **99**(17): 11493-500.

Ward, D. V., J. R. Zupan, et al. (2002). "*Agrobacterium* VirE2 gets the VIP1 treatment in plant nuclear import." Trends Plant Sci **7**(1): 1-3.

Ward, J. E., D. E. Akiyoshi, et al. (1988). "Characterization of the *virB* operon from an *Agrobacterium tumefaciens* Ti plasmid." J Biol Chem **263**(12): 5804-14.

Ward, J. E., D. E. Akiyoshi, et al. (1990). "Correction: characterization of the *virB* operon from *Agrobacterium tumefaciens* Ti plasmid." J Biol Chem **265**(8): 4768.

Ward, J. E., Jr., E. M. Dale, et al. (1991). "Activity of the *Agrobacterium* T-DNA transfer machinery is affected by *virB* gene products." Proc Natl Acad Sci U S A **88**(20): 9350-4.

Ward, J. E., Jr., E. M. Dale, et al. (1990). "Complementation analysis of *Agrobacterium tumefaciens* Ti plasmid *virB* genes by use of a *vir* promoter expression vector: *virB9*, *virB10*, and *virB11* are essential virulence genes." J Bacteriol **172**(9): 5187-99.

Ward, J. E., Jr., E. M. Dale, et al. (1990). "Identification of a *virB10* protein aggregate in the inner membrane of *Agrobacterium tumefaciens*." J Bacteriol **172**(9): 5200-10.

Watarai, M., H. L. Andrews, et al. (2001). "Formation of a fibrous structure on the surface of *Legionella pneumophila* associated with exposure of DotH and DotO proteins after intracellular growth." Mol Microbiol **39**(2): 313-29.

Weinert, L. A., J. H. Werren, et al. (2009). "Evolution and diversity of *Rickettsia* bacteria." BMC Biol **7**(1): 6.

Weisburg, W. G., M. E. Dobson, et al. (1989). "Phylogenetic diversity of the Rickettsiae." J Bacteriol **171**(8): 4202-6.

Wilgenbusch, J. C. and D. Swofford (2003). "Inferring evolutionary trees with PAUP*." Curr Protoc Bioinformatics **Chapter 6**: Unit 6 4.

Williams, K. P., B. W. Sobral, et al. (2007). "A robust species tree for the *Alphaproteobacteria*." J Bacteriol **189**(13): 4578-86.

Winans, S. C., D. L. Burns, et al. (1996). "Adaptation of a conjugal transfer system for the export of pathogenic macromolecules." Trends Microbiol **4**(2): 64-8.

Winans, S. C. and G. C. Walker (1985). "Conjugal transfer system of the IncN plasmid pKM101." J Bacteriol **161**(1): 402-10.

Winiecka-Krusnell, J. and E. Linder (1999). "Free-living amoebae protecting *Legionella* in water: the tip of an iceberg?" Scand J Infect Dis **31**(4): 383-5.

Winiecka-Krusnell, J. and E. Linder (2001). "Bacterial infections of free-living amoebae." Res Microbiol **152**(7): 613-9.

Winiecka-Krusnell, J., K. Wreiber, et al. (2002). "Free-living amoebae promote growth and survival of *Helicobacter pylori*." Scand J Infect Dis **34**(4): 253-6.

Yeo, H. J., S. N. Savvides, et al. (2000). "Crystal structure of the hexameric traffic ATPase of the *Helicobacter pylori* type IV secretion system." Mol Cell **6**(6): 1461-72.

Yeo, H. J. and G. Waksman (2004). "Unveiling molecular scaffolds of the type IV secretion system." J Bacteriol **186**(7): 1919-26.

Yeo, H. J., Q. Yuan, et al. (2003). "Structural and functional characterization of the VirB5 protein from the type IV secretion system encoded by the conjugative plasmid pKM101." Proc Natl Acad Sci U S A **100**(26): 15947-52.

Yuan, Q., A. Carle, et al. (2005). "Identification of the VirB4-VirB8-VirB5-VirB2 pilus assembly sequence of type IV secretion systems." J Biol Chem **280**(28): 26349-59.

Zahrl, D., M. Wagner, et al. (2005). "Peptidoglycan degradation by specialized lytic transglycosylases associated with type III and type IV secretion systems." Microbiology **151**(Pt 11): 3455-67.

Zupan, J., C. A. Hackworth, et al. (2007). "VirB1* promotes T-pilus formation in the *vir*-Type IV secretion system of *Agrobacterium tumefaciens*." J Bacteriol **189**(18): 6551-63.

Zupan, J., T. R. Muth, et al. (2000). "The transfer of DNA from *Agrobacterium tumefaciens* into plants: a feast of fundamental insights." Plant J **23**(1): 11-28.

# Appendix G: Type 4 Secretion System Named Entity Guidelines Annotation Guidelines

# Introduction

Type 4 secretion systems (T4SS) are bacterial mechanisms for transporting DNA and proteins into eukaryotic hosts. The secretion systems have been found in a range of bacteria, including *Agrobacterium tumefaciens, Helicobacter pylori, Bordetella pertussis and Legionella pneumophila.*  Named entities associated with T4SS are categorizes as organisms, genes/proteins, biological processes, cellular components, and molecular functions.

# Organisms

Organism names typically consist of a genus and species, such as:

- *Agrobacterium tumefaciens*
- *Bordetella pertussis*
- *Legionella pneumophila*

Genus names may be abbreviated to the first letter of the genus name, such as:

- *A. tumefaciens*
- *B. pertussis*
- *L. pneumophila*

Subspecies and strain names should be included in the named entity when available. The subspecies and strain name may be introduced with terms ‘subsp.’ and ‘str.’ Examples include:

- *Bacteroides melaninogenicus subsp. Intermedius*
- *Bordetella pertussis Tohama I*
- *Agrobacterium tumefaciens str. C58*

# Genes/Proteins

The gene and protein entities are tagged according to rules specified in:

Zhou Guo Dong, Shen Dan, Zhang Jie, Su Jian, Tan Soon Heng and Tan Chew Lim. Recognition of Protein and Gene Name Recognition from Text using an Ensemble of Classifiers and Effective Abbreviation Resolution. BMC Bioinformatics, 6(supp 1):S7, 24 May 2005, ISSN 1471-2105. [SCI Expanded]

# Biological Processes

The following are biological processes (as defined in the GO ontology) associated with Type 4 secretion systems:

- cell projection organization
- cell wall macromolecule catabolic process
- cellular response to stimulus
- cellular response to stress
- conjugation
- conjugation with cellular fusion
- defense response to bacterium
- pathogenesis
- peptidoglycan catabolic process
- pilus assembly
- protein secretion
- protein secretion by the type IV secretion system
- response to stimulus
- response to stress
- secretion by cell
- transport
- unidirectional conjugation

Some of these terms, such as “pilus assembly”, are routinely used in the literature; others, such as “conjugation with cellular fusion” are not often literally present. Concepts related to these terms are tagged as biological processes; examples include:

- transmission of DNA
- conjugal transfer of DNA
- protein secretion
- protein transport process
- subcellular localization
- T-complex transfer
- Plasmid transfer

Names of specific genes or plasmids are included in transfer processes. For example, in “…to accumulate free RP4 plasmid transfer intermediates …” the phrase “RP4 plasmid transfer” is tagged as a biological process. Similarly, in “ … low temperature stimulates the virB-dependent transfer of … “ the phrase “virB-dependent transfer” is tagged a biological process.

Other terms categorized as biological processes, in the appropriate contexts, include:

- localization
- translocation
- export
- conjugated
- virulence

# Cellular Components

The following are cellular components (as defined in the GO ontology) associated with Type 4 secretion systems:

- macromolecular complex
- protein complex
- type IV secretion system complex
- cell outer membrane
- integral to membrane
- membrane
- outer membrane
- plasma membrane
- periplasmic space

Some of these terms, such as “membrane”, are routinely used in the literature; others, such as “integral to membrane” are not often present in that literal form. Concepts related to these terms are tagged as cellular components; examples include:

- type IV secretion system
- transport systems
- bacterial envelope
- macromolecular transport systems

Other terms that are more specific to conjugation apparatus include:

- sex pilus
- conjugal pore
- bacterial conjugation apparatus

Specifically named cellular components and substrates are included in entity names tagged as cellular components. Examples include:

- VirA-VirG two-component transduction system
- T-complex transport apparatus
- IncQ substrates
- F-plasmid conjugation system

# Molecular Functions

The following are cellular components (as defined in the GO ontology) associated with Type 4 secretion systems:

- binding
- ATP binding
- nucleic acid binding
- DNA binding
- damaged DNA binding
- single-stranded DNA binding
- RNA binding
- nucleotide binding
- purine nucleotide binding
- hydrolase activity
- lysozyme activity
- nucleoside-triphosphatase activity
- helicase activity
- protein transporter activity

Some of these terms, such as “DNA binding”, are routinely used in the literature; others, such as “protein transporter activity” are not as frequently used in the literal form. Concepts related to these terms are tagged as cellular components; examples include:

- GTP-binding
- guanine-nucleotide-exchange activity
- GTPgS binding
- mononucleotide binding
- ATPase activity

The terms “activity” and “binding” can indicate a molecular function; those words are included in the terms tagged as molecular functions. Plural forms of these words, as in “ATPase activities” are also tagged as molecular function entities.
