# Supplementary material for: Named Entity Recognition for Bacterial Type IV Secretion Systems
Source: PLoS One. 2011 Mar 29;6(3):e14780. doi: 10.1371/journal.pone.0014780 (PMC3066171; doi:10.1371/journal.pone.0014780)
Supplement: Supporting Information S2 — Source document extracts and scripts. The results described in this paper can be recreated by following the workflow described in the file entitled "Instructions for Executing T4SS Named Entity Recognition Workflow.docx." (4.93 MB ZIP) [file pone.0014780.s002.zip › Revised Instructions and Data/Instructions for Executing T4SS Named Entity Recognition Workflow_w_Alan_edits_Dan_edits.docx]

# Instructions for Executing T4SS Named Entity Recognition Workflow

The results described in this paper can be recreated by following the workflow described below.

**Figure 1. High level overview of the steps required to generate T4SS NERs as described in “Named Entity Recognition for Bacterial Type IV Secretion Systems”, S. Ananiadou, D. Sullivan, et. al.**

## Step 1. Extracting Text from Literature

27 T4SS papers were used to train the named entity recognizers. For each paper, extract the text from the source and save as a text file. Presumably, experimenters will have access to the papers; not all papers used in this study are open access.

| **Source Reference** |
| --- |
| Baron, C., O. C. D, et al. (2002). "Bacterial secrets of secretion: EuroConference on the biology of type IV secretion processes." Mol Microbiol 43(5): 1359-65. |
| Beaupre, C. E., J. Bohne, et al. (1997). "Interactions between VirB9 and VirB10 membrane proteins involved in movement of DNA from Agrobacterium tumefaciens into plant cells." J Bacteriol 179(1): 78-89. |
| Berger, B. R. and P. J. Christie (1993). "The Agrobacterium tumefaciens virB4 gene product is an essential virulence protein requiring an intact nucleoside triphosphate-binding domain." J Bacteriol 175(6): 1723-34. |
| Blanc, G., H. Ogata, et al. (2007). "Lateral gene transfer between obligate intracellular bacteria: evidence from the Rickettsia massiliae genome." Genome Res 17(11): 1657-64. |
| Cao, T. B. and M. H. Saier, Jr. (2001). "Conjugal type IV macromolecular transfer systems of Gram-negative bacteria: organismal distribution, structural constraints and evolutionary conclusions." Microbiology 147(Pt 12): 3201-14. |
| Christie, P. J. (1997). "Agrobacterium tumefaciens T-complex transport apparatus: a paradigm for a new family of multifunctional transporters in eubacteria." J Bacteriol 179(10): 3085-94. |
| Christie, P. J. and J. P. Vogel (2000). "Bacterial type IV secretion: conjugation systems adapted to deliver effector molecules to host cells." Trends Microbiol 8(8): 354-60. |
| Citovsky, V., A. Kapelnikov, et al. (2004). "Protein interactions involved in nuclear import of the Agrobacterium VirE2 protein in vivo and in vitro." J Biol Chem 279(28): 29528-33. |
| Covacci, A., J. L. Telford, et al. (1999). "Helicobacter pylori virulence and genetic geography." Science 284(5418): 1328-33. |
| Finberg, K. E., T. R. Muth, et al. (1995). "Interactions of VirB9, -10, and -11 with the membrane fraction of Agrobacterium tumefaciens: solubility studies provide evidence for tight associations." J Bacteriol 177(17): 4881-9. |
| Guyon, P., M. D. Chilton, et al. (1980). "Agropine in "null-type" crown gall tumors: Evidence for generality of the opine concept." Proc Natl Acad Sci U S A 77(5): 2693-2697. |
| Jakubowski, S. J., E. Cascales, et al. (2005). "Agrobacterium tumefaciens VirB9, an outer-membrane-associated component of a type IV secretion system, regulates substrate selection and T-pilus biogenesis." J Bacteriol 187(10): 3486-95. |
| Jones, A. L., K. Shirasu, et al. (1994). "The product of the virB4 gene of Agrobacterium tumefaciens promotes accumulation of VirB3 protein." J Bacteriol 176(17): 5255-61. |
| Medini, D., A. Covacci, et al. (2006). "Protein homology network families reveal step-wise diversification of Type III and Type IV secretion systems." PLoS Comput Biol 2(12): e173. |
| Nagai, H., J. C. Kagan, et al. (2002). "A bacterial guanine nucleotide exchange factor activates ARF on Legionella phagosomes." Science 295(5555): 679-82. |
| Ogata, H., S. Audic, et al. (2000). "Selfish DNA in protein-coding genes of Rickettsia." Science 290(5490): 347-50. |
| Roy, C. R. and L. G. Tilney (2002). "The road less traveled: transport of Legionella to the endoplasmic reticulum." J Cell Biol 158(3): 415-9. |
| Segal, E. D., J. Cha, et al. (1999). "Altered states: involvement of phosphorylated CagA in the induction of host cellular growth changes by Helicobacter pylori." Proc Natl Acad Sci U S A 96(25): 14559-64. |
| Segal, G., J. J. Russo, et al. (1999). "Relationships between a new type IV secretion system and the icm/dot virulence system of Legionella pneumophila." Mol Microbiol 34(4): 799-809. |
| Stephens, K. M., C. Roush, et al. (1995). "Agrobacterium tumefaciens VirB11 protein requires a consensus nucleotide-binding site for function in virulence." J Bacteriol 177(1): 27-36. |
| Tzfira, T. and V. Citovsky (2002). "Partners-in-infection: host proteins involved in the transformation of plant cells by Agrobacterium." Trends Cell Biol 12(3): 121-9. |
| Tzfira, T., M. Vaidya, et al. (2004). "Involvement of targeted proteolysis in plant genetic transformation by Agrobacterium." Nature 431(7004): 87-92. |
| Ward, D. V., J. R. Zupan, et al. (2002). "Agrobacterium VirE2 gets the VIP1 treatment in plant nuclear import." Trends Plant Sci 7(1): 1-3. |
| Ward, J. E., D. E. Akiyoshi, et al. (1988). "Characterization of the virB operon from an Agrobacterium tumefaciens Ti plasmid." J Biol Chem 263(12): 5804-14. |
| Ward, J. E., D. E. Akiyoshi, et al. (1990). "Correction: characterization of the virB operon from Agrobacterium tumefaciens Ti plasmid." J Biol Chem 265(8): 4768. |
| Weisburg, W. G., M. E. Dobson, et al. (1989). "Phylogenetic diversity of the Rickettsiae." J Bacteriol 171(8): 4202-6. |
| Williams, K. P., B. W. Sobral, et al. (2007). "A robust species tree for the Alphaproteobacteria." J Bacteriol 189(13): 4578-86. |

**Table 1. Source files used in T4SS NER study.**

## Step 2: Apply Sentence Splitter and Part of Speech (POS) Taggers

For each text file extracted in Step 1, apply a sentence splitter and part of speech tagger to the text, to produce a two column, one token per line output. The Genia tagger, freely available at http://www-tsujii.is.s.u-tokyo.ac.jp/GENIA/tagger/.

The script rungenia.sh , included in the in our supplemental materials: T4SS/scripts/rungenia.sh can be used for this, as follows:

Before rungenia.sh the first time, edit the script and change the two shell variables GENIA_HOME to be the directory that you build genia in, and SPLITTER_LIB to absolute path of the jar file T4SS/lib/sptoolkit.jar. Then run:

T4SS/scripts/rungenia.sh < fileN.txt > fileN.genia

The script's prerequisites are a Java virtual machine to run the Splitter. Instructions on installing the

Genia tagger are available at <http://www-tsujii.is.s.u-tokyo.ac.jp/GENIA/tagger/>.

Typical output from the Genia tagger would look like

| A DT  Bacterial NNP  Guanine NNP  Nucleotide NNP  Exchange NNP  Factor NNP |
| --- |

## Step 3: Annotate a set of documents to indicate entities in the source material, to be used to train the entity recognizer.

Each line of the output of the genia tagger needs to be annotated with a either “B” to indicate the beginning of an entity name “I” to indicate subsequent words are included in the term, and “O” to indicate the word is not part of an entity name.

A sample article that has been so annotated is the T4SS/data/originals/file1_first_paragraph.txt For reasons of copyright, for the rest of the articles, only those lines that are annotated “B” and “I”. In order to use these annotated files as input to the subsequent steps to reproduce our work, you need to acquire the full text of the article, run the tagger, and merge the corresponding files in the directory T4SS/data/extracted, filling in “O” on lines that that are not include in the extracted files.

## Step 4: Run the computeFeatures.py Script

The output files from Step 2, e.g. fileN.genia, are used as input to computeFeatures.py to produce the feature set used by the CRF to train the NERs. The Python script computeFeatures.py is included with the Supplementary Material T4SS/scripts/.

The arguments to computeFeatures .py are: an input file, a name of an output file, dictionary 1 file name, dictionary 2 file name, and mode. All dictionary files used with this experiment are included in the Supplementary Material.

As there are no desired secondary properties, for the experiments in this paper, the first and the second dictionaries are the same file. They are located in dict/ folder. The last argument called mode is to set the appropriate tag whilst building the features. For example, if the model was for cell part, we want the dictionary to be called CC rather than BP or MF. Depending on the classifier, the mode is changed to one of the CC(dictionaries/go_subset_cell_part_ids.txt ), MF(dictionaries/go_subset_molecular_function_ids.txt ), BP(dictionaries/go_subset_biological_process_ids.txt ), or BACT(dictionaries/all_bact_ncbi_lpsn._3col.txt) .

The training will be done separately for each type of entity. In each case a single file is created that is the concatenation of all the output files of the previous step, for inputs annotated for the type of entity. Assuming that the file that is the concatenation of the outputs from cellular component inputs is called all_cc_files.txt. Features for the CRF model for the type CC (in this example) are generated by using the computeFeatures.py script.

Assuming one is in the supplemental materials directory T4SS, execute

python scripts/computeFeatures.py all_cc_files.txt all_cc_features dictionaries/go_subset_cell_part_ids.txt dictionaries/go_subset_cell_part_ids.txt CC

The output file will include features describing the pre and post context of each word, the part of speech, and orthology features. An example output for from the computeFeatures.py script is:

Guanine Guanin CC_0 ORG_0 NNP wf_Aaaaaaa first_G wffirst_A wflastfour_aaaa wflasttwo_aa lastfour_nine lasttwo_ne PRE_1_Bacterial PRE_ORG_1_1 PRE_CC_1_1 PRE_POS_1_NNP PRE_wf_1Aaaaaaaaa PRE_first_1_B PRE_wffirst_1_A  PRE_wflastfour_1_aaaa PRE_wflasttwo_1_aa PRE_lastfour_1_rial PRE_lasttwo_1_al PRE_2_A PRE_ORG_2_0 PRE_CC_2_0 PRE_POS_2_DT PRE_wf_2A PRE_first_2_A PRE_wffirst_2_A  PRE_wflastfour_2_A PRE_wflasttwo_2_A PRE_lastfour_2_A PRE_lasttwo_2_A POST_1_Nucleotide POST_ORG_1_0 POST_CC_1_1 POST_POS_1_NNP POST_wf_1Aaaaaaaaaa POST_first_1_N POST_wffirst_1_A  POST_wflastfour_1_aaaa POST_wflasttwo_1_aa POST_lastfour_1_tide POST_lasttwo_1_de POST_2_Exchange POST_ORG_2_0 POST_CC_2_1 POST_POS_2_NNP POST_wf_2Aaaaaaaa POST_first_2_E POST_wffirst_2_A  POST_wflastfour_2_aaaa POST_wflasttwo_2_aa POST_lastfour_2_ange POST_lasttwo_2_ge POST_3_Factor POST_ORG_3_1 POST_CC_3_1 POST_POS_3_NNP POST_wf_3Aaaaaa POST_first_3_F POST_wffirst_3_A  POST_wflastfour_3_aaaa POST_wflasttwo_3_aa POST_lastfour_3_ctor POST_lasttwo_3_or O

## Step 5. Train the CRF to Posit Model

## The CRF program, Mallet-0.4 is used in this experiment to implements the conditional random field algorithm. Mallet software is open source under Open Source Initiative (OSI) Common Public License Version 1.0. The Conditional Random Field implementation is available from http://mallet.cs.umass.edu. Documentation is available from

http://mallet.cs.umass.edu/sequences.php.

Download <http://mallet.cs.umass.edu/dist/mallet-0.4.tar.gz> and unpack it into the T4SS/lib directory.

The Mallet-0.4 program is invoked using output from the previous step using a command such as the following (all on one line)

java -cp T4SS/lib//mallet-0.4/lib/\* edu.umass.cs.mallet.base.fst.SimpleTagger
--train true 
--gaussian-variance 100.0
--model-file T4SS_cc.model all_cc_features

This will create a model called T4SS_cc.model that can be used to recognize cell part terms in new files.

## Step 6. Use the Classifier on Test Data

## The output of Step 4 is a model which can be applied to test data. A subset of the 27 annotated files may be excluded from use in training (Step 4) and used here in Step 5 as test data. The Mallet-0.4 program is for this step but the –train parameter is set to false to execute the model

java -cp T4SS/lib/mallet-0.4/lib/\*
edu.umass.cs.mallet.base.fst.SimpleTagger
--train false
--model-file T4SS_cc.model
testData

The output of this program will be a single character per line per line of input, encoding which terms or sets of terms constitute a entity of the type. For example given the input

The DT
intracellular JJ
pathogen NN
Legionella NN
pneumophila NN
subverts NNS
vesicle NN

The output

O
O
O
B CC
I -CC
O
O

Indicates that there is a single entity recognized that begins with the word “Legionella” indicated by the code “B” and includes the word “pneumophila” indicated by the “I”, i.e. the term “Legionella pneumophila”
